# Oxytocin and vasopressin modulation of prisoner’s dilemma strategies

**DOI:** 10.1177/0269881120913145

**Published:** 2020-03-24

**Authors:** Maria Leonor Neto, Marília Antunes, Manuel Lopes, Duarte Ferreira, James Rilling, Diana Prata

**Affiliations:** 1 Faculty of Psychology and Neuroscience, Maastricht University, Maastricht, The Netherlands; 2 Centro de Estatística e Aplicações e Departamento de Estatística e Investigação Operacional, Faculdade de Ciências, Universidade de Lisboa, Lisbon, Portugal; 3 INESC-ID, Instituto Superior Técnico, Lisbon, Portugal; 4 Instituto de Medicina Molecular, Faculdade de Medicina da Universidade de Lisboa, Lisbon, Portugal; 5 Department of Anthropology, Emory University, Atlanta, USA; 6 Department of Neuroimaging, Institute of Psychiatry, Psychology and Neuroscience, King’s College London, London, UK; 7 Instituto de Biofísica e Engenharia Biomédica, Faculdade de Ciências da Universidade de Lisboa, Lisbon, Portugal; 8 Centro de Investigação e Intervenção Social (CIS-IUL), Instituto Universitário de Lisboa (ISCTE-IUL), Lisbon, Portugal

**Keywords:** Neuropeptides, game theory, cooperation, competition, theory of mind, cognitive empathy, prisoner’s dilemma, mentalising

## Abstract

**Background:**

The neuropeptides oxytocin and vasopressin have been repeatedly implicated in social decision making by enhancing social salience and, generally, cooperation. The iterated and sequential version of the prisoner’s dilemma (PD) game is a social dilemma paradigm eliciting strategies of cooperation versus competition.

**Aims:**

We aimed to characterise the role of PD players’ sex, game partner type (computer vs. human) and oxytocin or vasopressin inhalation on the player’s strategy preference.

**Methods:**

Participants (153 men; 151 women) were randomised to intranasal 24 IU oxytocin, 20 IU vasopressin or placebo, double-blind, and played the PD. We examined main and interactive effects of sex, drug and partner type on strategy preference.

**Results:**

We found a pervasive preference for a tit-for-tat strategy (i.e. general sensitivity to the partner’s choices) over unconditional cooperation, particularly when against a human rather than a computer partner. Oxytocin doubled this sensitivity in women (i.e. the preference for tit-for-tat over unconditional cooperation strategies) when playing against computers, which suggests a tendency to anthropomorphise them, and doubled women’s unconditional cooperation preference when playing against humans. Vasopressin doubled sensitivity to the partner’s previous choices (i.e. for tit-for-tat over unconditional cooperation) across sexes and partner types.

**Conclusions:**

These findings suggest that women may be more sensitive to oxytocin’s social effects of anthropomorphism of non-humans and of unconditional cooperation with humans, which may be consistent with evolutionary pressures for maternal care, and that vasopressin, irrespective of sex and partner type, may be generally sensitising humans to others’ behaviour.

## Introduction

Predicting others’ intentions (i.e. mentalising) is essential for building trust and achieving cooperation (Brüne and Brüne-Cohrs, 2006). In most cases, cooperation is rewarding and reinforces trust by favourably updating our mentalisation of who treats us fairly. Recent findings on the biochemical basis of social decision making point to neuropeptides oxytocin and vasopressin as key modulators of the neural circuitry supporting it, such as in trust, cooperation or revenge. Nevertheless, the influence of both systems on the strategies adopted to solve social dilemmas, and how they depend on the player’s sex and partner type, remains unclear.

Oxytocin modulates various social behaviours. In animals, it influences social decision making (Meyer-Lindenberg et al., 2011), social salience (Meyer-Lindenberg et al., 2011), attachment, maternal nurturing and stress resilience (Ferris, 2005; Heinrichs et al., 2009). Vasopressin shares affinity for the same receptors and is a mediator of attachment, social recognition and aggression (Ferris, 2005; Heinrichs et al., 2009). In humans, pharmacological studies have demonstrated oxytocin’s role in social cognition in healthy subjects and in psychiatric patients (Ishak et al., 2011; Meyer-Lindenberg et al., 2011). For example, intranasal oxytocin increases the perceived trustworthiness of faces (Theodoridou et al., 2009), improves the accuracy of mental-state inferences (De Dreu et al., 2011; Domes et al., 2007; Guastella et al., 2010), enhances learning from social cues (Hurlemann et al., 2010) and seems to increase conformity (De Dreu and Kret, 2016; Xu et al., 2019), particularly in a competitive context (Aydogan et al., 2017). In game theory tasks, it increases generosity (Barraza et al., 2011; Zak et al., 2007), cooperation (Declerck et al., 2010, 2013; Ditzen et al., 2009) and trust (Bakermans-Kranenburg and Van IJzendoorn, 2013; Kosfeld et al., 2005), although not consistently replicated (Nave et al., 2015), while vasopressin has increased mutual cooperation (Brunnlieb et al., 2016). There is some evidence that oxytocin’s (and possibly vasopressin’s) prosocial effects are limited to in-group members (De Dreu et al., 2010, 2011) and that vasopressin has antisocial effects in humans, such as selfish and punitive behaviour (Stanton, 2007).

**Figure 1.**
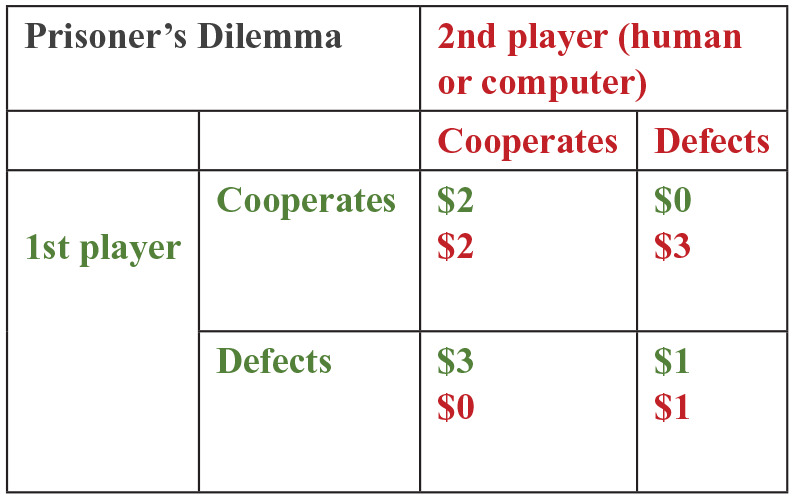
Representation of the pay-off matrix for the prisoner’s dilemma game. Players 1 and 2 can either cooperate or defect, and afterwards the pay-off for the round is represented by the square specified by the two decisions. In green (upper value) is the pay-off for player 1, and in red (lower value) the pay-off for player 2.

The prisoner’s dilemma (PD) is a paradigm that emphasises the tension between the collectively highest (thus welfare-maximising) outcome for both players and the individually pay-off-maximising outcome. The earliest occurrence of the term PD dates back to 1950, coined by Albert W. Tucker (Luce and Raiffa, 1957), while the game is thought to have been devised by Merril Flood and Melvin Dresher (Poundstone, 1993). The game has been extensively studied by game theorists. In the single-shot version, two players simultaneously and independently choose either to cooperate or to defect. Each cell of the pay-off matrix (see Figure 1) indicates the pay-off for each player, given their choices. While mutual cooperation is often associated with friendship, love, trust or obligation, mutual defection relates to feelings of rejection or hatred. Jointly, the cooperator typically feels anger or indignation, and the defector feels anxiety, guilt or elation from successfully exploiting the partner (Rilling et al., 2012). In this one-off simultaneous choice version, mutual defection is the dominant strategy, that is, the Nash equilibrium (Osborne and Rubinstein, 1999). Computer simulations of the iterated version of the PD, where the same game is played several times, have included strategies such as, in descending order of cooperativeness: Cooperator (Coop), Tit-for-Two-Tats (TF2T), Tit-for-Tat (TFT) and Defector (Def). The TFT takes place when the player mimics, in the current round, the partner’s choice in the previous one. In TF2T, when the player chooses to cooperate and the partner chooses to defect, the player only plays ‘defect’ after two consecutive ‘cooperate-defect’ outcomes. In the Coop and Def strategies, the player simply cooperates or defects, respectively, in every round, irrespective of what the partner has played in the previous rounds. In the iterated version of the game, the maximum gain for both players combined occurs from *mutual* cooperation, but this outcome is unstable (i.e. each player has an incentive to defect in order to gain an extra benefit in the current round). Among these strategies, TFT has been the most successful because it is ‘nice’ (i.e. never first to defect) and retaliatory yet forgiving: ironically, for a strategy to maximise benefit, it must involve some forgiveness (Axelrod and Hamilton, 1981).

Extrapolating to ecological contexts, TFT is thought to be an evolutionarily stable strategy (i.e. resistant to invasion by other strategies; Easley and Kleinberg, 2010; Gintis, 2000). It leads to high fitness in social species, provided that individuals interact sufficiently often and that there is an initial drive to cooperate (Axelrod and Hamilton, 1981). There are several examples of TFT in nature (Ferris, 2005). In species where TFT is observed, this initial drive towards cooperation might have originated between kin, allowing for a genetically transmitted self-reinforcing bias. ‘Kin’-like altruistic behaviour between unrelated individuals (within-species) is observed in some social animals (e.g. egg trading in hermaphroditic fish (Crowley and Hart, 2007) and food sharing among vampire bats (Wilkinson, 1988)). Mutualism (between-species) also exists (e.g. in house sparrows (Dugatkin, 2002), primates (Dugatkin, 2002), and in chimpanzees interacting with humans (Warneken et al., 2007)). Reciprocal altruism or direct reciprocity (i.e. the expectation that cooperation will be rewarded with cooperation) has also been demonstrated in both primates (De Waal and Luttrell, 1988) and bats (Dugatkin, 2002; Wilkinson, 1988). Cooperation towards unrelated strangers also has evolutionary explanations such as sexual selection, whereby displays of wealth and generosity are regarded as attractive traits (Zahavi, 1975, 1995); and indirect reciprocity, whereby helping others may increase one’s reputation (Nowak and Sigmund, 2005).

We have previously reported the effects of intranasal oxytocin and vasopressin, player sex and partner type (human vs. computer) on behaviour, and their neural correlates during the iterated and sequential-choice PD game. Using the same (Chen et al., 2016; Feng et al., 2015) and part of the same (Rilling et al., 2012, 2014) sample herein, we found that both intranasal oxytocin and vasopressin had different effects in male and female participants. In a subsample of 91 men (Rilling et al., 2012), we found that, when playing with human partners, oxytocin increased rates of cooperation following unreciprocated cooperation compared to vasopressin, whereas vasopressin increased rates of cooperation following cooperation from human and computer partners. In a sample of 87 women (Rilling et al., 2014), we found drug effects exclusively with computer partners: oxytocin lowered the probability of cooperating following unreciprocated cooperation or mutual defection, and vasopressin lowered it following mutual defection. In these cases, both neuropeptides lowered rates of cooperation with computer partners, which may reflect anthropomorphism (i.e. attribution of social meaning to inanimate stimuli). In an enlarged sample of 153 men and 151 women, we have also reported that vasopressin treatment decreased the probability of cooperating after unreciprocated cooperation from computer partners, but only in men (Chen et al., 2016).

Given the above-mentioned evidence that oxytocin and, less consistently, vasopressin increase trust and cooperation, one prediction is that treatment with these neuropeptides will bias subjects towards the most cooperative PD strategy (Coop) and away from the least cooperative strategy (Def). On the other hand, the social salience hypothesis predicts that oxytocin and vasopressin sensitise players to their partner’s behaviour, rendering their cooperation more contingent on their partner’s choice, creating a bias towards TFT or TF2T.

To evaluate these two possibilities, we analysed behavioural responses of the first player (see Methods) during the iterated and sequential version of the PD in a double-blind placebo-controlled study in which either oxytocin or vasopressin was administered. We have previously reported (Chen et al., 2016; Feng et al., 2015; Rilling et al., 2014) on PD outcome frequency and transition probabilities (e.g. probability of cooperating after mutual cooperation (Feng et al., 2015) or after unreciprocated cooperation (Chen et al., 2016)). In the present analysis, we assessed whether these neuropeptides modulate preference for various PD strategies based on a trial-by-trial pattern of responses. This overcomes the limitation in our previous work and, in the majority of the literature, of reporting average responses, disregarding the sequence of outcomes. To this end, we selected the above-mentioned iterated strategies (Coop, TF2T, TFT and Def) and calculated, through maximum-likelihood, a strategy for each participant, after which we inferred how strategy preference depended on drug treatment, sex and partner type.

## Methods

### Sample and drug administration

A sample of 153 men and 151 women from the Emory University community between the ages of 18 and 22 years (men *M*_age_=20.7 years; women *M*_age_=20.5 years) was recruited and randomised to self-administer 10 nasal puffs of one of the following: 24 IU of intranasal oxytocin (*n*=50 for both men and women; Syntocinin-Spray; Novartis, Basel Switzerland), 20 IU of intranasal vasopressin (*n*=49 for men and *n*=51 for women; American Reagent Laboratories, Shirley,) or intranasal placebo (*n*=54 for men and *n*=50 for women), during a functional magnetic resonance imaging scanning session. (At the time of collection this was the most common dose used, producing measurable cognitive behavioural effects; MacDonald et al., 2011). Subjects were told they would receive oxytocin, vasopressin or placebo, and were instructed to place the nasal applicator in one nostril and depress the lever until they felt a mist of spray in the nostril, then to breathe in deeply through the nose, and afterwards to place the applicator in the other nostril and repeat the process. The present sample is included in previous reports which follow the same protocol (Chen et al., 2016; Feng et al., 2015; Rilling et al., 2012, 2014).

All subjects gave written informed consent, and the study was approved by the Emory University Institutional Review Board.

### The prisoner’s dilemma task

In the PD game, two players choose either to cooperate or to defect and receive a pay-off that depends upon the interaction of their respective choices. The game version we use here is the above-mentioned iterated version of the PD, where the same game is played several times with the same partner, and is sequential, in which player 1 chooses and player 2 is then able to view player 1’s choice before making his/her own choice. This game serves as a model for relationships based on trust and reciprocity (Axelrod and Hamilton, 1981; Chen et al., 2016; Rilling et al., 2012; Yao et al., 2014). Player 1 must decide whether to trust player 2 (i.e. cooperate), whereas player 2 must decide whether to reciprocate cooperation (or defection). Each of the four game outcomes is associated with a different pay-off. Player cooperation followed by partner cooperation (CC) pays US$2 to both player and partner; player cooperation followed by partner defection (CD) pays US$0 to the player and US$3 to the partner; player defection followed by partner defection (DD) pays US$1 to both player and partner; and player defection followed by partner cooperation (DC) pays US$3 to the player and US$0 to the partner (see Figure 1). As described in our previous reports (Chen et al., 2016; Feng et al., 2015), subjects played 30 rounds of an iterated PD game with the putative human partner (who was represented by a same-sex confederate who had previously been briefly introduced to him/her), and another 30 with a computer partner (in counterbalanced order). In reality, subjects were playing with a preprogramed computer algorithm in both sessions. The algorithm strategy was designed to mimic an actual human strategy: it reciprocated defection at 90% and cooperation at 67%. After the experiment, participants were paid two-thirds of their total earnings.

### Subject-level strategy preference identification

We compared each subject’s game choices, as player 1, to known iterated PD game strategies, namely TFT, TF2T, Coop and Def. TFT, which involves a relatively lower level of forgiveness and trust than TF2T, has been considered one of the most successful strategies in the iterated (and sequential) PD (Axelrod and Hamilton, 1981). Here, we aimed to contrast this optimal strategy with: (a) Coop, which involves the highest level of forgiveness and trust; (b) TF2T, which involves a higher level of forgiveness and trust (than TFT); and (c) the Def strategy, which involves the lowest level of forgiveness and trust (thus, higher level of fear of betrayal and defensiveness). We also contrasted the latter three between themselves.

The strategy preferred by each individual (as player 1) was identified using the maximum-likelihood method. For a given strategy, we calculated which action is expected to be executed. For instance, when following a TFT strategy, we expect that the subject defects (D) in a given round after suffering a defection in the previous one. By considering that for each strategy there is always a well-defined action, we can define a probability function that assigns a high probability (*p*_H_=0.95) if the correct (i.e. the expected) action was executed, and a low probability (*p*_L_=0.05) otherwise. For instance, in the case of a Coop strategy, we would assign a probability of 0.95 to a cooperation (C) choice and 0.05 to a D choice, regardless of the outcome of the previous round. In the case of a TFT strategy, we would assign a probability of 0.95 to a C choice following a DC or CC round and to a D choice following a DD or a CD round; and would assign a probability of 0.05 to the remaining possible outcome combinations. Then, we normalise the resulting likelihood by dividing the un-normalised likelihoods of each strategy by their total sum.

All of the 30 round-specific likelihood values were applied a logarithmic transformation and then summed in order to obtain an estimate of how the player’s choices across all 30 rounds compared to each of the included strategies, and the model’s equation is described in Supplement 1. In the end, the strategy showing the highest likelihood (according to the subject’s game choices) was chosen as that subject’s strategy. This procedure resulted in two outcomes for each subject, since the partner variable (human, computer) is a within-subjects variable. (Further details are available in Supplement 1.)

### Group-level strategy preference comparisons

For the group analysis, a general estimating equation approach was used to estimate a logistic multinomial regression model using a logit link in R studio v1.0.153 (R Foundation for Statistical Computing, Vienna, Austria). In these models, one of the strategies is used as reference strategy (in this case, TFT), and three model equations are estimated, allowing each of the remaining strategies to be compared to the reference strategy. The dependent variable was ‘strategy preference’. Independent between-subject variables were drug (oxytocin, vasopressin, placebo) and sex (male, female), and the independent within-subject variable was partner (human, computer), which entered the model as sets of dummy variables. All corresponding effects and possible interactions were estimated, and the model’s equation is described in Supplement 1, along with further details on its design.

To estimate the main effects (of drug, partner and sex), we considered three simple models of the same class as described above, considering each of the independent variables alone. To estimate the two-way interaction effects (of drug×partner, drug×sex and sex×partner), we considered three models of the same type of the above-mentioned, with all of the possible pairs of factors. We considered a trend any effect showing a *p*-value of <0.10, and a statistically significant effect any effect showing a *p*-value of <0.05.

## Results

### Overall strategy preference

**Figure 2.**
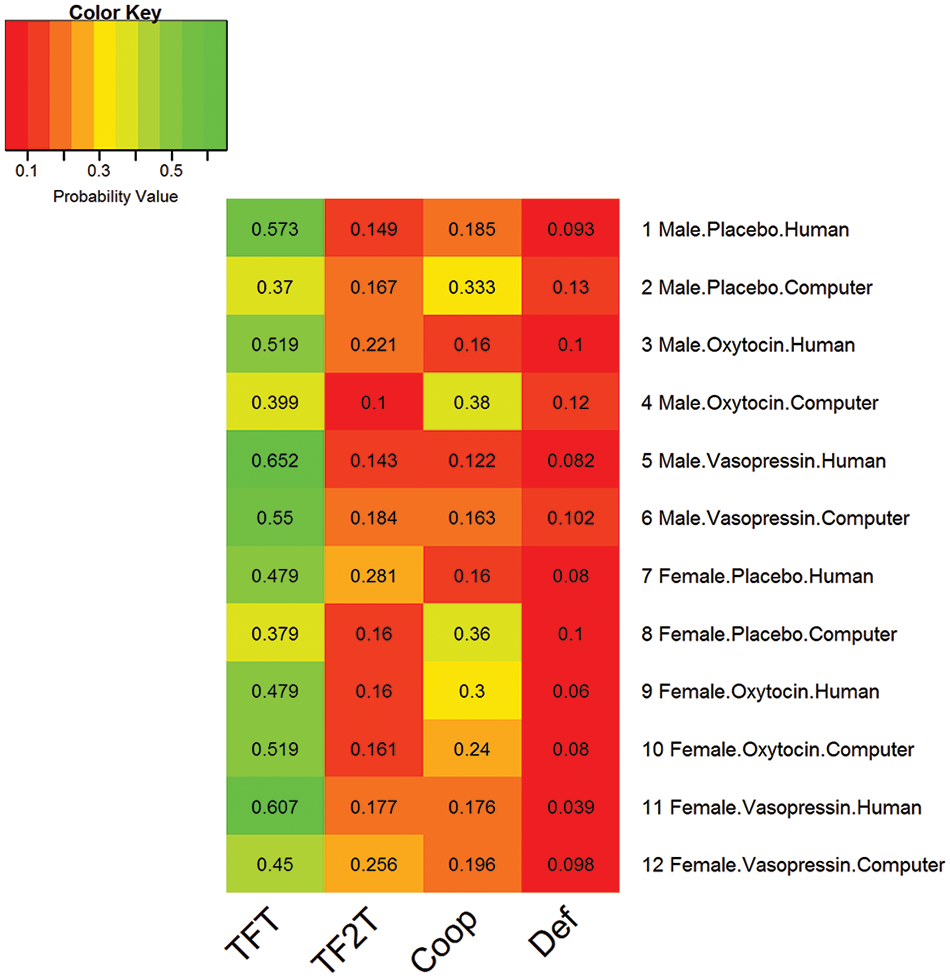
Heat map of the probability of choosing each of the four possible strategies (in descending order of cooperativeness – Cooperator (Coop), Tit-for-Two-Tats (TF2T), Tit-for-Tat (TFT) and Defector (Def) – in each smallest homogeneous group of subjects in terms of sex, drug and partner. TFT was the preferred strategy (~50%), then Coop (~23%), then TF2T (~18%) and then Def (~9%).

Across all 608 sessions and subjects, TFT was by far the preferred strategy (*n*=303; nearly 50%) across the different groups (see Figure 2), followed by Coop (*n*=139; nearly 23%) and TF2T (*n*=113; nearly 18%), with Def being the least preferred strategy (*n*=53; nearly 9%).

**Figure 3.**
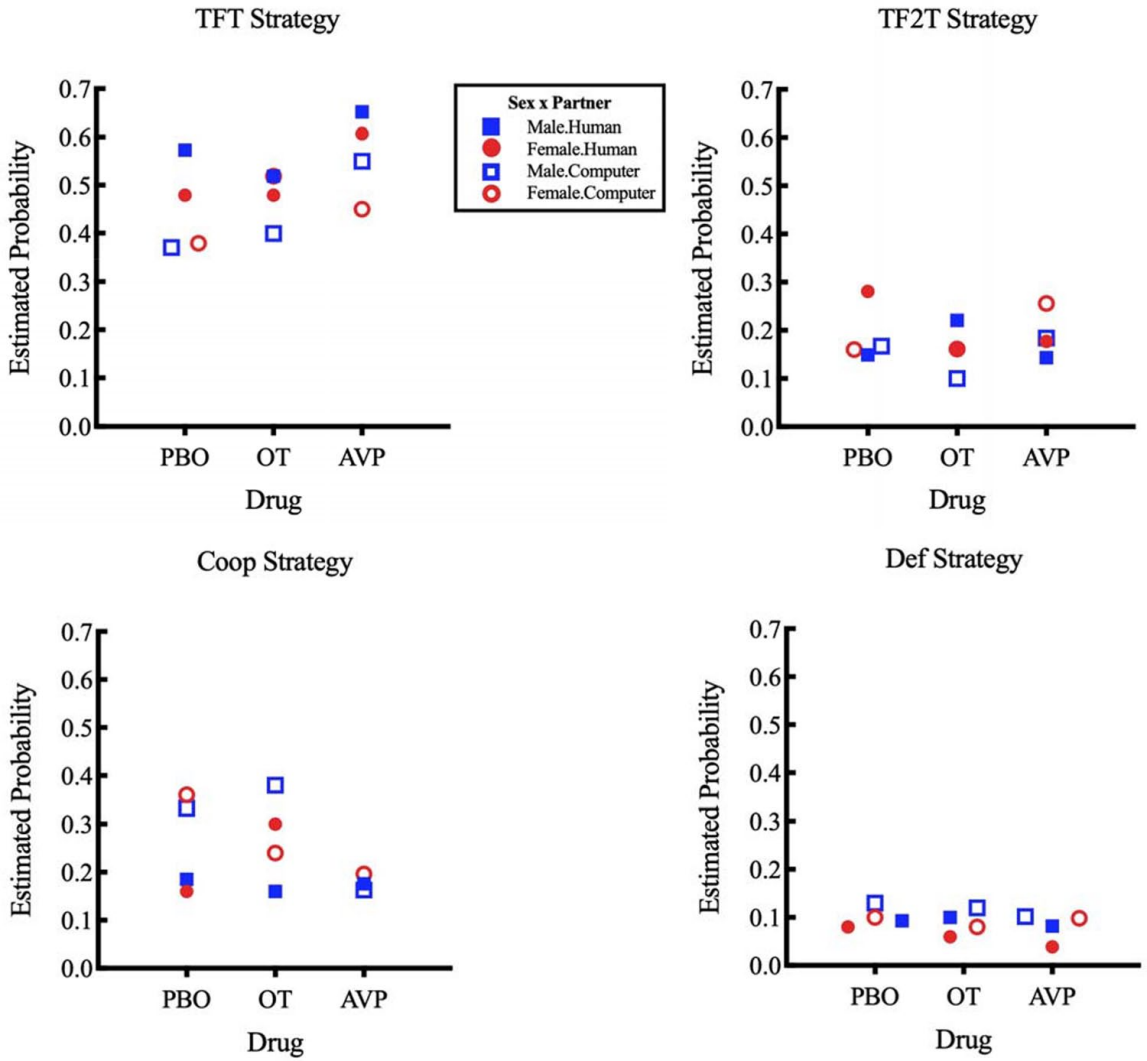
Estimated probability of choosing a given strategy in terms of sex, drug and partner for (a) TFT, (b) TF2T, (c) Coop and (d) Def. PBO: placebo; OT: oxytocin; AVP: vasopressin.

The strategy preference probabilities for each of the 12 possible subject profiles, considering sex, drug and partner, are presented in a heat map (see Figure 2) and in graphical format (see Figure 3). By calculating ratios of these probabilities, we can infer how more strongly preferred a strategy is compared to others as a function of our variables of interest (sex, drug and partner). In particular, we calculated how the preference between pairs of strategies varied as a function of sex, drug and partner. In the following sections, we report pairs of strategies for which we found significant main effects: two- or three-way interactions. The preference for TFT over Coop and the preference for TF2T over Coop was influenced by our three variables of interest (sex, drug and partner) as we show below (for further details on those, and results for the complete list of ratios, including TFT/TF2T, TFT/Def, TF2T/Def and Coop/Def, see Supplement 2, Figures S1–S4).

### TFT over Coop preference

**Figure 4.**
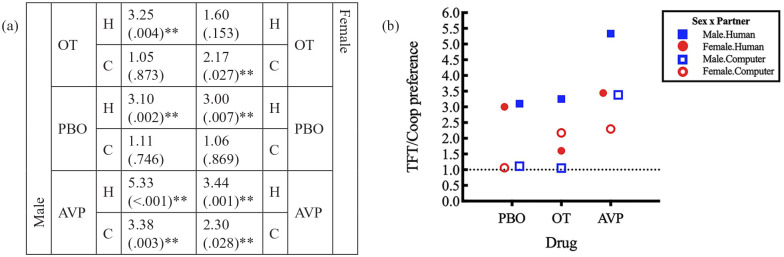
Preference for TFT over Coop and respective *p*-values as a function of sex, drug and partner. Estimate values >1 present a preference for TFT, while estimates <1 point to a preference for Coop (**p<0.05; *p>0.1) in (a) table format and (b) graphical format. Regarding drug effects, the following main effects and interactions were found: a three-way sex×drug (PBO vs. OT)×partner interaction (*p*=0.076); for oxytocin takers, a sex×partner interaction (*p*=.007); for female players, a drug (PBO vs. OT)×partner interaction (*p*=0.018); and a main effect of drug (PBO vs. AVP); *p*=0.022; see Supplement 2, Tables S1, S3 and S7).

We found a trend for a sex×drug×partner three-way interaction (*p*=0.076; Supplement 2, Table S1, oxytocin.computer.female) on the TFT strategy over Coop strategy preference. For male players, there was a significant preference for TFT over Coop under both placebo and oxytocin when they played with a human partner (3.10 times for placebo takers, *p*=0.002; 3.25 times for oxytocin takers, *p*=0.004; Figure 4(a)). In both treatments, this preference was not found when they played with a computer partner. Placebo or oxytocin administration did not affect the difference in preference as a function of partner type (for male placebo takers, the ratio of preferences between human and computer was 3.10/1.11=2.79; for male oxytocin takers, this same ratio was 3.25/1.05=3.10, giving a non-significant difference; see Figure 4(a)).

On the other hand, for female players, we found a significant difference in the preference for TFT over Coop, characterised by an interaction between drug and partner (*p*=0.018; Supplement 2, Table S7, oxytocin.computer). For female players under placebo, there was a significant preference for TFT over Coop when they played with a human partner (3.00, *p*=0.007; Figure 4(a)) that disappeared when they played with a computer partner (the ratio of preference is thus 3.00/1.06=2.83; see Figure 4(a)). That is, they behaved as male placebo takers. However, when under the influence of oxytocin, this preference was reversed, which was characterised by an interaction between drug and sex (*p*=0.007; Supplement 2, Table S3, computer.female). When women under oxytocin played with a human partner, there was no preference between the two strategies, but when playing against a computer partner, they significantly preferred TFT over Coop (2.17 times, *p*=0.027; Figure 4(a)).

The above also emerged as a highly significant main effect of partner, as shown by a decrease of 47% in the preference for TFT over Coop when the partner was a computer (3.00×0.53=1.59 times, *p*<0.001; Supplement 2, Table S14) versus a human (3.00 times; Supplement 2, Table S14).

As for taking placebo versus vasopressin and oxytocin versus vasopressin, no significant differences were found neither for males nor females. Still, we found a significant main effect of drug, which emerged as an increase in the preference for TFT over Coop of nearly twofold (1.97 times, *p*=0.022; Supplement 2, Table S15) when comparing the use of vasopressin with the use of placebo, which resulted in a pervasive significant preference for TFT over Coop for males and females under vasopressin, regardless of partner type (see Figure 4(a) for preferences and *p*-values associated with each profile).

### TF2T over Coop preference

**Figure 5.**
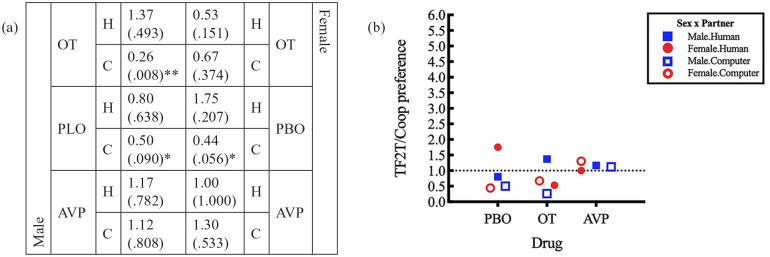
Preference for TF2T over Coop and respective *p*-values as a function of sex, drug and partner. Estimate values >1 present a preference for TF2T, while estimates <1 point to a preference for Coop (**p<0.05; *p>0.1) in (a) table format and (b) graphical format. Drug wise, the following interactions were found: a three-way sex×drug (PBO vs. OT)×partner interaction (*p*=0.024); for a human partner, a sex×drug interaction (PBO vs. OT; *p*=0.057); for oxytocin takers, a sex×partner interaction (*p*=0.041); for female players, a drug (PBO vs. OT)×partner interaction (*p*=0.069); and for female players, a drug (PBO vs. AVP)×partner interaction (*p*=0.042; see Supplement 2, Tables S1, S4, S7 and S9).

We found a significant three-way sex×drug×partner interaction (*p*=0.024; Supplement 2, Table S1, oxytocin.computer.female) on the TF2T over Coop strategy preference, in the same direction as the above TFT versus Coop results. For male players, there was a trend for a preference for Coop over TF2T under placebo and a significant preference for Coop over TF2T when they were playing against a computer partner (0.5 times preference of TF2T over Coop for placebo takers, *p*=0.090; 0.26 times preference of TF2T over Coop for oxytocin takers, *p*=0.008; Figure 5(a)). This preference was not found when playing with a human partner. As such, in men, taking placebo or taking oxytocin did not affect the difference in preference caused by changing partner (for male placebo takers, the ratio of preferences between human and computer was 0.80/0.50=1.60; for male oxytocin takers, this same ratio was 1.37/0.26=5.27, which was a non-significant difference; see Figure 5(a)).

On the other hand, for female players, we found a significant difference in the preference for TF2T over Coop characterised by an interaction trend between drug and partner (*p*=0.069; Supplement 2, Table S7, oxytocin.computer). For female players under placebo, there was a trend for a preference for Coop over TF2T when they played against a computer (0.44 times, *p*=0.056; Figure 5(a)) that disappeared when they played against a human (the ratio of preference is thus 1.75/0.44=4.00; see Figure 5(a)). That is, they behaved as male placebo takers. But when they were under the influence of oxytocin, this preference disappeared (the ratio is 0.53/0.67=0.79), which was characterised by a trend for a sex×drug (placebo vs. oxytocin) interaction for a human partner (*p*=0.057; Supplement 2, Table S7, oxytocin.male) and also a significant sex×partner interaction for oxytocin takers (*p*=0.041; Supplement 2, Table S9, computer.male). That is, oxytocin rendered women more likely to always cooperate. Additionally, we found a drug (placebo vs. vasopressin)×partner interaction for female players (*p*=0.042; Supplement 2, Table S7, vasopressin.computer). That is, for female players under vasopressin, there was no preference between the two strategies. They behaved as male players under vasopressin and female players under oxytocin, contrary to female players under placebo, for whom there was a difference in the preference.

The above also emerged as a significant main effect of partner, as shown by a 40% drop in the preference for TF2T over Coop when the partner was a computer (1.02×0.60=0.61, *p*=0.041; Supplement 2, Table S14) versus a human (1.02; Supplement 2, Table S14). No further interactions were found to be significant (but see Figure 5(a) for preferences and *p*-values associated with each profile).

### Main effects

For completeness, we further summarise all statistically significant main effects. In respect to drug effects, we found a main effect of drug on the TFT over Coop preference, with vasopressin leading to a nearly twofold (i.e. 1.97) increase in TFT preference (*p*=0.022; Supplement 2, Table S15; see Figure 4(b) AVP column for a visual depiction). There was a main effect of partner on the TFT over Coop preference, which dropped 47% when subjects played against a computer versus a human partner (*p*<0.001; Supplement 2, Table S14). The same effect was found for the TFT over Def preference (drop of 42%, *p*=0.029) and for the TF2T over Coop preference (drop of 40%, *p*=0.04; Supplement 2, Table S14). In the latter case, as opposed to the former cases, there was no strategy preference when playing against a human, but a significant preference for Coop when playing against a computer.

## Discussion

We sought to characterise the impact of intranasal oxytocin, vasopressin, player sex and partner type on strategy choice during a social dilemma. We found that the preference for tit-for-tat strategies (TFT and TF2T) over unconditional cooperation was affected by all of the above factors.

### Overall social strategy preference

In general, we found that tit-for-tat strategies were preferred over unconditional cooperation (Coop) or defection (Def) when playing against humans (Supplement 2, Table S14), which was expected, given that they have been considered to be more evolutionarily stable than the latter strategies (Axelrod, 1980; Axelrod and Hamilton, 1981). There was a decrease of 40% for TF2T and 47% for TFT in preference over unconditional cooperation when subjects played with a computer, which expectedly indicates that playing with humans entices higher (social) salience to the actions of others. In addition, TFT was preferred over TF2T in almost every group (except females under placebo playing against humans or under vasopressin playing against computers; Supplement 2, Figure S1).

### Overall drug effects (and interactions) on social strategy preference

With respect to drug effects of social strategy preference, our present results, discussed next in detail, suggest that oxytocin effects show substantial dependence on the subject’s sex and the partner’s human agency. In women, they are consistent both with the prosocial hypothesis of oxytocin, since oxytocin increased trust and cooperation with humans, and with the social salience hypothesis of oxytocin, since oxytocin increased contingency on others’ past behaviour, from non-humans. As vasopressin also increased contingency on others’ past behaviour – from humans and non-humans and in both sexes – a vasopressin social salience hypothesis is suggested, but a prosocial hypothesis is not supported.

We found that intranasal oxytocin increased cooperative behaviour among women (decreasing the relative preference for both tit-for-tat strategies) while they played with a supposedly human partner. Specifically, influencing the TFT over unconditional cooperation preference, we found: (a) a significant main effect of partner type; (b) a significant main effect of drug for vasopressin; and (c) a highly significant influence of sex on partner preference within oxytocin takers only (which translated into a three-way interaction trend). That is, men preferred TFT when playing against humans, showing no preference between strategies when playing against computers, and this did not change with oxytocin intake. Women taking placebo showed the same behaviour as men (towards both humans and computers), but oxytocin intake increased their preference for unconditional cooperation when playing against humans and for TFT when playing against computers (Figure 4, discussed below).

#### General increased sensitivity to human versus computer behaviour

The finding that both sexes, under placebo, showed a decreased preference for a TFT strategy (vs. unconditional cooperation) and, conversely, an increased preference for unconditional cooperation when playing against computers (vs. humans) may indicate that we tend not to retaliate defections from computers as much as from other humans. This may mean that a tendency towards revenge, or fear of a second defection, is present when playing against humans but not against computers, perhaps because no intentionality or deliberation is attributed to a computer (i.e. it is playing randomly), and therefore a defection is not considered a breach of trust. Another non-mutually exclusive explanation is that no learning is attributed to the computer partner, and thus the corrective punishment involved in a tit-for-tat strategy would have no effect. On the contrary, when a defection from the partner is thought to be deliberate and intentional, as in the case of a human partner, there is a breach of trust or a defensive fear of re-defection. Thus, a punishing retaliation might increase partner cooperation.

#### Oxytocin’s facilitation of sensitivity to computer behaviour and of unconditional cooperation with humans in women

Importantly, as stated, oxytocin altered the above effect in women (i.e. it caused an increase in preference for unconditional cooperation over TFT when playing against humans vs. computers), but not in men. In women, oxytocin caused the computer-induced change in preference towards unconditional cooperation (and away from TFT) not to occur. With oxytocin, when playing against a computer, women exhibited the same preference that both sexes had shown for a tit-for-tat strategy when playing against a human under placebo, which leads us to hypothesise that women with heightened oxytocin function treated the computer as a human, that is, they anthropomorphised the computer. As such, due to oxytocin intake, women may have employed a more revengeful, defensive or corrective attitude towards the computer as if it had an intentional attitude. Such an oxytocin facilitation of anthropomorphisation in women has indeed been reported for socially moving geometric shapes (Scheele et al., 2015), although not consistently (Hecht et al., 2017). Moreover, the same oxytocin intake in women decreased this revengeful/punitive/defensive attitude when they played against perceived humans. Thus, a heightened oxytocin function seems to increase women’s levels of cooperation with humans by rendering it less contingent on the other player’s choice. In fact, it is possible that oxytocin enhances women’s learning that cooperation is the most rewarding strategy of this game (which is ‘taught’ by the automated (second player) tit-for-tat-resembling algorithm that punishes defection and rewards cooperation moves).

The above suggests that men are less sensitive than women to oxytocin in terms of its effect on strategy choices at the 24 IU oxytocin dose. Women may be more lenient than men in social interactions when experiencing higher oxytocin function, herein mimicked by intranasal intake. This is plausibly consistent with evolutionary pressures towards making women more tolerable to children’s aversive behaviour during childcare, given previous findings that oxytocin plasma levels during pregnancy and the first month postpartum correlate with higher levels of behavioural and psychological bonding with the infants (Feldman et al., 2007), and that oxytocin may be released to reduce stress and anxiety in mothers with low sensitivity and ability to cope with their infants, thus promoting care and bonding (Elmadih et al., 2014). Not excluding the explanation proposed above for an increase in cooperation, oxytocin may also be enhancing fear of the consequences of not collaborating, specifically in women. Indeed, a number of studies have shown that oxytocin effects on amygdala neural responses to negative affective stimuli tend to be increased in women but decreased in men (Gao et al., 2016; Lischke et al., 2012), which is further supported by a sex-dependent receptor distribution in the amygdala (in non-primate mammals; Francis et al., 2002; Stoop, 2012).

#### Converging oxytocin evidence from both tit -for-tat strategies

Serving as converging evidence, our TF2T versus Coop comparison showed broadly the same effect direction as the above-mentioned TFT versus Coop comparison. Indeed, both tit-for-tat strategies (TFT and TF2T) are expected to contrast with unconditional cooperation in the same direction, although TF2T is somewhat intermediate between TFT and Coop, as it involves a relatively higher level of trust and forgiveness (i.e. cooperation) than TFT. Here, we also found a main effect of partner type, revealing an increase in the preference for Coop when playing against a computer opponent. The three-way interaction was stronger and statistically significant in the TF2T (rather than TFT) versus Coop case. Analogous to the TFT versus Coop comparison (see above), in the TF2T versus Coop case, men, irrespective of drug, as well as women under placebo, showed a tendency to cooperate unconditionally with computers but not with humans. Again, these tendencies, and especially the latter with humans, disappeared in women under intranasal oxytocin, which suggests that oxytocin hampers cooperative attitude towards computers in women, and increases it towards humans. This implies that oxytocin heightened a revengeful/punitive/defensive behaviour towards computers. Lastly, the modulating effect of sex on the effect of oxytocin also survived, as a trend, irrespective of partner type. Interestingly, oxytocin administration aligned all four groups in the same order in terms of preference for tit-for-tat strategies versus unconditional cooperation (Figures 4 and 5).

#### Comparison with previous (average-based) oxytocin findings

The above results (TFT and TF2T vs. Coop) corroborate and expand our previous conclusion, reported with part of the present sample (~59%), that oxytocin lowers the cooperative attitude with computers following a mutual defection outcome or an unreciprocated cooperation outcome in women, but not as much in men (Rilling et al., 2012, 2014). However, using the exact same sample, we also reported a null effect of oxytocin and of any interaction with sex on the probability of cooperating following a mutual cooperation (Feng et al., 2015), on the number of cooperation choices and on the probability of cooperating following unreciprocated cooperation (Chen et al., 2016). This suggests that analyses using transition probabilities may be insufficiently sensitive to the effects of these drugs.

Others have reported (Yao et al., 2014) that women show more punitive behaviour than men do towards humans who breach their trust – which, in the present case, is what we found for computer but not for human partners. On the other hand, it has also been previously reported (Scheele et al., 2014) in another moral dilemma task that women under oxytocin exhibit more prosocial behaviour, endorsing in less self-benefit outcomes, whereas men under oxytocin show a bias towards self-benefiting outcomes. This result may further converge with the disappearance of a preference for TFT over unconditional cooperation when women under oxytocin played against a human (vs. a computer). At the same time, evidence (Theodoridou et al., 2013) suggests that oxytocin enhances social perspective taking in men, having no effect in women, perhaps because, in women, this heightened tendency is already present, regardless of oxytocin intake. However, in the present study, oxytocin did not change the preference in men, but only in women. Lastly, the present results are consistent with both the social salience hypothesis of oxytocin and the prosocial hypothesis of oxytocin by providing converging evidence that it increases generosity, cooperation, trust and anthropomorphism in women.

#### Vasopressin’s general facilitation of sensitivity to the behaviour of others

As for vasopressin, we found that it increased the preference for a TFT strategy over unconditional cooperation nearly twofold, irrespective of sex or partner. This difference was not noticeable in the TF2T versus Coop comparison. These findings partially converge with our previous transition probability results (Rilling et al., 2012, 2014). Using part of the present sample (~59%), we showed that vasopressin increased conciliatory behaviour in women (i.e. increased the probability of cooperation following unreciprocated cooperation), but also increased reciprocation of cooperation when men played against both computers and humans. Among women, vasopressin lowered the rates of cooperation with computers following an outcome of mutual defection in the previous round. Even though this effect was seen exclusively in women playing against a computer and does not amount directly to a TFT strategy, it is in accordance with what we see in the present analysis. Furthermore, we also reported, with the present sample and data, that vasopressin increased the probability of cooperating after a mutual cooperation more in men than in women (Feng et al., 2015) and that it decreased the probability that men would cooperate after an unreciprocated cooperation outcome from computers (Chen et al., 2016), which agrees with the pervasive preference for a TFT strategy.

Overall, the present findings show that vasopressin generally renders subjects’ behaviour more contingent on partners’ choice, irrespective of sex or partner type. Even though the present results diverge from the idea that vasopressin increases mutual cooperation (Brunnlieb et al., 2016), they converge with previous findings that suggest that vasopressin can have antisocial effects, such as selfish and punitive behaviour (Stanton, 2007). Furthermore, this effect might be attributable to vasopressin increasing the salience of social stimuli (e.g. the partner’s choice).

### Limitations and future directions

As a reflection on potential limitations, the present analysis is based on assigning a preferred strategy to each individual playing against a specific partner through maximum-likelihood, which differs from our previous analysis that compared transition probabilities (Chen et al., 2016; Feng et al., 2015; Rilling et al., 2012, 2014). As such, comparisons between both types of data should be made with caution. There is also still limited research in this field including sex as a quasi-experimental variable, which is warranted given previous findings that point to distinct neural substrates between the sexes (Rilling et al., 2014). Additionally, until there is evidence to show that playing against players of the same or different sex can modulate the effect of oxytocin or vasopressin, we cannot be sure the drug-level difference we found is exclusively due to the participant’s sex and not to the partner’s. Nevertheless, in the present study, the participants always played against those of the same sex. There is indeed evidence that opposite-sex dyads result in different levels of cooperation, namely more competition in same-sex dyads (Mack et al., 1971), and that in opposite-sex dyads there is decreased sensitivity to the opponent’s behaviour and strategy (Knight, 1980). However, given that previous research has mostly focused on studying same-sex dyads, we too opted to study same-sex dyads to facilitate comparability with the literature on this social dilemma.

Further studies should explore if the dose of oxytocin alters the observed pattern of behaviour, namely to investigate support for the inverted U-shape hypothesis of social reward (Borland et al., 2018). This hypothesis proposes that there is an inverted U-shape relationship between oxytocin dose, social reward and neural activity in males and females. This hypothesis is still rather unexplored in humans. However, these and related findings on the effect of these neuropeptides in human behaviour may contribute to the better design of both aetiological and intranasal oxytocin therapeutic models of social symptoms – particularly in anxiety, psychosis and autism spectrum disorders.

## Conclusion

In sum, oxytocin increased the proportion of women who adopted the ‘always cooperate’ strategy with human partners (and reduced it when with computers, therein approaching men’s behaviour). In men, oxytocin produced no effect, and they were less cooperative, more retaliatory and more corrective with human compared to non-human partners. Women may be more lenient than men in social interactions when experiencing higher oxytocin function, herein mimicked by intranasal intake, which is plausibly consistent with evolutionary pressures towards making women more tolerable to children’s aversive behaviour during childcare, which in turn is well aligned with the heightened physiological measurements of oxytocin in women during maternal bonding. Furthermore, vasopressin intake increased the proportion of subjects who played a tit-for-tat strategy, where cooperation is more contingent on the partner’s choice. That is, vasopressin may be sensitising players to their partner’s choices.

Overall, our findings improve the knowledge of the biological mechanisms involved in social/economic decision making in humans and, importantly, emphasise sex differences in a literature where mostly men are researched. By doing so, they may help better explain, physiologically, the different behavioural deficit patterns seen between sexes, in the same psychiatric illnesses, for example in antisocial personality disorder or autism spectrum disorder, where women tend to show less social deficits and be more prosocial. Clinically, they can also help predict the behavioural effects of using these neuropeptides therapeutically, as is being trialled nowadays in autism spectrum disorders and schizophrenia.

## Supplemental Material

Supplement_1_JPsychopharm_REVISED – Supplemental material for Oxytocin and vasopressin modulation of prisoner’s dilemma strategies Click here for additional data file. Supplemental material, Supplement_1_JPsychopharm_REVISED for Oxytocin and vasopressin modulation of prisoner’s dilemma strategies by Maria Leonor Neto, Marília Antunes, Manuel Lopes, Duarte Ferreira, James Rilling and Diana Prata in Journal of Psychopharmacology

Supplement_2_JPsychopharm – Supplemental material for Oxytocin and vasopressin modulation of prisoner’s dilemma strategies Click here for additional data file. Supplemental material, Supplement_2_JPsychopharm for Oxytocin and vasopressin modulation of prisoner’s dilemma strategies by Maria Leonor Neto, Marília Antunes, Manuel Lopes, Duarte Ferreira, James Rilling and Diana Prata in Journal of Psychopharmacology

## References

[bibr1-0269881120913145] Axelrod R (1980) Effective choice in the prisoner’s dilemma. J Confl Resolut 24: 3–25.

[bibr2-0269881120913145] Axelrod R Hamilton WD (1981) The evolution of cooperation. Science 211: 1390–1396 7466396 10.1126/science.7466396

[bibr3-0269881120913145] Aydogan G Jobst A D’Ardenne K, et al (2017) The detrimental effects of oxytocin-induced conformity on dishonesty in competition. Psychol Sci 28: 751–759. 28388303 10.1177/0956797617695100

[bibr4-0269881120913145] Bakermans-Kranenburg MJ Van IJzendoorn MH (2013) Sniffing around oxytocin: review and meta-analyses of trials in healthy and clinical groups with implications for pharmacotherapy. Transl Psychiatry 3: e258. 10.1038/tp.2013.34 PMC3669921 23695233

[bibr5-0269881120913145] Barraza JA McCullough ME Ahmadi S, et al (2011) Oxytocin infusion increases charitable donations regardless of monetary resources. Horm Behav 60: 148–151. 21596046 10.1016/j.yhbeh.2011.04.008

[bibr6-0269881120913145] Borland JM Rilling JK Frantz KJ, et al (2019) Sex-dependent regulation of social reward by oxytocin: an inverted U hypothesis. Neuropsychopharmacology 44: 97–110. 29968846 10.1038/s41386-018-0129-2 PMC6235847

[bibr7-0269881120913145] Brüne M Brüne-Cohrs U (2006) Theory of mind – evolution, ontogeny, brain mechanisms and psychopathology. Neurosci Biobehav Rev 30: 437–455. 16239031 10.1016/j.neubiorev.2005.08.001

[bibr8-0269881120913145] Brunnlieb C Nave G Camerer CF, et al (2016) Vasopressin increases human risky cooperative behavior. Proc Natl Acad Sci U S A 113: 2051–2056. 26858433 10.1073/pnas.1518825113 PMC4776476

[bibr9-0269881120913145] Chen X Hackett PD DeMarco AC, et al (2016) Effects of oxytocin and vasopressin on the neural response to unreciprocated cooperation within brain regions involved in stress and anxiety in men and women. Brain Imaging Behav 10: 581–593. 26040978 10.1007/s11682-015-9411-7 PMC4670292

[bibr10-0269881120913145] Crowley PH Hart MK (2007) Evolutionary stability of egg trading and parceling in simultaneous hermaphrodites: the chalk bass revisited. J Theor Biol 246: 420–429. 17335851 10.1016/j.jtbi.2007.01.018

[bibr11-0269881120913145] De Dreu CKW Kret ME (2016) Oxytocin conditions intergroup relations through upregulated in-group empathy, cooperation, conformity, and defense. Biol Psychiatry 79: 165–173. 25908497 10.1016/j.biopsych.2015.03.020

[bibr12-0269881120913145] De Dreu CKW Greer LL Handgraaf MJJ, et al (2010) The neuropeptide oxytocin regulates parochial altruism in intergroup conflict among humans. Science 328: 1408–1411. 20538951 10.1126/science.1189047

[bibr13-0269881120913145] De Dreu CKW Greer LL Van Kleef GA, et al (2011) Oxytocin promotes human ethnocentrism. Proc Natl Acad Sci U S A 108: 1262–1266. 21220339 10.1073/pnas.1015316108 PMC3029708

[bibr14-0269881120913145] De Waal FB Luttrell LM (1988) Mechanisms of social reciprocity in three primate species: symmetrical relationship characteristics or cognition? Evol Hum Behav 9: 101–118.

[bibr15-0269881120913145] Declerck CH Boone C Kiyonari T (2010) Oxytocin and cooperation under conditions of uncertainty: the modulating role of incentives and social information. Horm Behav 57: 368–374. 20080100 10.1016/j.yhbeh.2010.01.006

[bibr16-0269881120913145] Declerck CH Boone C Kiyonari T (2013) The effect of oxytocin on cooperation in a prisoner’s dilemma depends on the social context and a person’s social value orientation. Soc Cogn Affect Neurosci 9: 802–809. 23588271 10.1093/scan/nst040 PMC4040087

[bibr17-0269881120913145] Ditzen B Schaer M Gabriel B, et al (2009) Intranasal oxytocin increases positive communication and reduces cortisol levels during couple conflict. Biol Psychiatry 65: 728–731. 19027101 10.1016/j.biopsych.2008.10.011

[bibr18-0269881120913145] Domes G Heinrichs M Michel A, et al (2007) Oxytocin improves ‘mind-reading’ in humans. Biol Psychiatry 61: 731–733. 17137561 10.1016/j.biopsych.2006.07.015

[bibr19-0269881120913145] Dugatkin LA (2002) Cooperation in animals: an evolutionary overview. Biol Philos 17: 459–476.

[bibr20-0269881120913145] Easley D Kleinberg J (2010) Networks, crowds, and markets: reasoning about a highly connected world. Science 81: 744.

[bibr21-0269881120913145] Elmadih A Wai M Numan M, et al (2014) Does oxytocin modulate variation in maternal caregiving in healthy new mothers? Brain Res 1580: 143–150. 24462937 10.1016/j.brainres.2014.01.020

[bibr22-0269881120913145] Feldman R Weller A Zagoory-Sharon O, et al (2007) Evidence for a neuroendocrinological foundation of human affiliation: plasma oxytocin levels across pregnancy and the postpartum period predict mother-infant bonding. Psychol Sci 18: 965–970. 17958710 10.1111/j.1467-9280.2007.02010.x

[bibr23-0269881120913145] Feng C Hackett PD DeMarco AC, et al (2015) Oxytocin and vasopressin effects on the neural response to social cooperation are modulated by sex in humans. Brain Imaging Behav 9: 754–764. 25416642 10.1007/s11682-014-9333-9

[bibr24-0269881120913145] Ferris CF (2005) Vasopressin/oxytocin and aggression. Novartis Found Symp 268: 190–198; discussion 198–200, 242–253. 16206881

[bibr25-0269881120913145] Francis DD Young LJ Meaney MJ, et al (2002) Naturally occurring differences in maternal care are associated with the expression of oxytocin and vasopressin (V1a) receptors: gender differences. J Neuroendocrinol 14: 349–353. 12000539 10.1046/j.0007-1331.2002.00776.x

[bibr26-0269881120913145] Gao S Becker B Luo L, et al (2016) Oxytocin, the peptide that bonds the sexes also divides them. Proc Natl Acad Sci U S A 113: 7650–7654. 27325780 10.1073/pnas.1602620113 PMC4941426

[bibr27-0269881120913145] Gintis H (2000) Game theory evolving. Ecol Econ 39: 479–480.

[bibr28-0269881120913145] Guastella AJ Einfeld SL Gray KM, et al (2010) Intranasal oxytocin improves emotion recognition for youth with autism spectrum disorders. Biol Psychiatry 67: 692–694. 19897177 10.1016/j.biopsych.2009.09.020

[bibr29-0269881120913145] Hecht EE Robins DL Gautam P, et al (2017) Intranasal oxytocin reduces social perception in women: neural activation and individual variation. Neuroimage 147: 314–329. 27989775 10.1016/j.neuroimage.2016.12.046

[bibr30-0269881120913145] Heinrichs M Von Dawans B Domes G (2009) Oxytocin, vasopressin, and human social behavior. Front Neuroendocrinol 30: 548–557. 19505497 10.1016/j.yfrne.2009.05.005

[bibr31-0269881120913145] Hurlemann R Patin A Onur OA, et al (2010) Oxytocin enhances amygdala-dependent, socially reinforced learning and emotional empathy in humans. J Neurosci 30: 4999–5007. 20371820 10.1523/JNEUROSCI.5538-09.2010 PMC6632777

[bibr32-0269881120913145] Ishak WW Kahloon M Fakhry H (2011) Oxytocin role in enhancing well-being: a literature review. J Affect Disord 130: 1–9. 20584551 10.1016/j.jad.2010.06.001

[bibr33-0269881120913145] Knight GP (1980) Behavioral similarity, confederate strategy, and sex composition of dyad as determinants of interpersonal judgments and behavior in the prisoner’s dilemma game. J Res Pers 14: 91–103.

[bibr34-0269881120913145] Kosfeld M Heinrichs M Zak PJ, et al (2005) Oxytocin increases trust in humans. Nature 435: 673–677. 15931222 10.1038/nature03701

[bibr35-0269881120913145] Lischke A Gamer M Berger C, et al (2012) Oxytocin increases amygdala reactivity to threatening scenes in females. Psychoneuroendocrinology 37: 1431–1438. 22365820 10.1016/j.psyneuen.2012.01.011

[bibr36-0269881120913145] Luce DR Raiffa H (1957) Games and Decisions: Introduction and Critical Survey. New York: John Wiley.

[bibr37-0269881120913145] MacDonald E Dadds MR Brennan JL, et al (2011) A review of safety, side-effects and subjective reactions to intranasal oxytocin in human research. Psychoneuroendocrinology 36: 1114–1126. 21429671 10.1016/j.psyneuen.2011.02.015

[bibr38-0269881120913145] Mack D Auburn PN Knight GP (1971) Sex role identification and behavior in a reiterated prisoner’s dilemma game. Psychon Sci 24: 280–282.

[bibr39-0269881120913145] Meyer-Lindenberg A Domes G Kirsch P, et al (2011) Oxytocin and vasopressin in the human brain: social neuropeptides for translational medicine. Nat Rev Neurosci 12: 524–538. 21852800 10.1038/nrn3044

[bibr40-0269881120913145] Nave G Camerer C McCullough M (2015) Does oxytocin increase trust in humans? A critical review of research. Perspect Psychol Sci 10: 772–789. 26581735 10.1177/1745691615600138

[bibr41-0269881120913145] Nowak MA Sigmund K (2005) Evolution of indirect reciprocity. Nature 437: 1291–1298. 16251955 10.1038/nature04131

[bibr42-0269881120913145] Osborne MJ Rubinstein A (1999) A Course in Game Theory. Cambridge, MA: MIT Press.

[bibr43-0269881120913145] Poundstone W (1993) Prisoner’s Dilemma. New York: Anchor Books.

[bibr44-0269881120913145] Rilling JK DeMarco AC Hackett PD, et al (2012) Effects of intranasal oxytocin and vasopressin on cooperative behavior and associated brain activity in men. Psychoneuroendocrinology 37: 447–461. 21840129 10.1016/j.psyneuen.2011.07.013 PMC3251702

[bibr45-0269881120913145] Rilling JK DeMarco AC Hackett PD, et al (2014) Sex differences in the neural and behavioral response to intranasal oxytocin and vasopressin during human social interaction. Psychoneuroendocrinology 39: 237–248. 24157401 10.1016/j.psyneuen.2013.09.022 PMC3842401

[bibr46-0269881120913145] Scheele D Schwering C Elison JT, et al (2015) A human tendency to anthropomorphize is enhanced by oxytocin. Eur Neuropsychopharmacol 25: 1817–1823. 26092202 10.1016/j.euroneuro.2015.05.009

[bibr47-0269881120913145] Scheele D Striepens N Kendrick KM, et al (2014) Opposing effects of oxytocin on moral judgment in males and females. Hum Brain Mapp 35: 6067–6076. 25094043 10.1002/hbm.22605 PMC6868938

[bibr48-0269881120913145] Stanton AA (2007) Neural substrates of decision-making in economic games. J Diss 1: 1–63.

[bibr49-0269881120913145] Stoop R (2012) Neuromodulation by oxytocin and vasopressin. Neuron 76: 142–159. 23040812 10.1016/j.neuron.2012.09.025

[bibr50-0269881120913145] Theodoridou A Rowe AC Mohr C (2013) Men perform comparably to women in a perspective taking task after administration of intranasal oxytocin but not after placebo. Front Hum Neurosci 7: 1–11. 23754995 10.3389/fnhum.2013.00197 PMC3664327

[bibr51-0269881120913145] Theodoridou A Rowe AC Penton-Voak IS, et al (2009) Oxytocin and social perception: oxytocin increases perceived facial trustworthiness and attractiveness. Horm Behav 56: 128–132. 19344725 10.1016/j.yhbeh.2009.03.019

[bibr52-0269881120913145] Warneken F Hare B Melis AP, et al (2007) Spontaneous altruism by chimpanzees and young children. PLoS Biol 5: 1414–1420. 10.1371/journal.pbio.0050184 PMC1896184 17594177

[bibr53-0269881120913145] Wilkinson GS (1988) Reciprocal altruism in bats and other mammals. Ethol Sociobiol 9: 85–100.

[bibr54-0269881120913145] Xu L Becker B Kendrick KM (2019) Oxytocin facilitates social learning by promoting conformity to trusted individuals. Front Neurosci 13: 56. 30787864 10.3389/fnins.2019.00056 PMC6372972

[bibr55-0269881120913145] Yao S Zhao W Cheng R, et al (2014) Oxytocin makes females, but not males, less forgiving following betrayal of trust. Int J Neuropsychopharmacol 17: 1785–1792. 24916520 10.1017/S146114571400090X

[bibr56-0269881120913145] Zahavi A (1975) Mate selection – a selection for a handicap. Reproduction 53: 205–214. 10.1016/0022-5193(75)90111-3 1195756

[bibr57-0269881120913145] Zahavi A (1995) Altruism as a handicap – the limitations of kin selection and reciprocity. J Avian Biol 26: 1–3.

[bibr58-0269881120913145] Zak PJ Stanton AA Ahmadi S (2007) Oxytocin increases generosity in humans. PLoS One 2: e1128. 10.1371/journal.pone.0001128 PMC2040517 17987115

