# Supplementary material for: Oxytocin and vasopressin modulation of prisoner’s dilemma strategies
Source: J Psychopharmacol. 2020 Mar 24;34(8):891–900. doi: 10.1177/0269881120913145 (PMC7583454; doi:10.1177/0269881120913145)
Supplement: Supplement_1_JPsychopharm_REVISED – Supplemental material for Oxytocin and vasopressin modulation of prisoner’s dilemma strategies [file Supplement_1_JPsychopharm_REVISED.pdf]

Supplement 1 - Methods

(with Further Detail relative to the Manuscript)

Subject-level strategy preference identification

We compared each subject's game choices, as Player 1, to known iterated PD game strategies, namely the Tit-for-Tat (TFT), Tit-for-Two-Tats (TF2T), Cooperator (Coop) and Defector (Def). TFT, which involves a relatively lower level of forgiveness and trust than TF2T, has been considered one of the most successful strategy in the iterated (and sequential) PD (Axelrod and Hamilton, 1981). Here, we aimed to contrast this optimal strategy with: 1) Coop, which involves the highest level of forgiveness and trust; 2) TF2T, which involves a higher level of forgiveness and trust (than TFT); and 3) the Def strategy, which involves the lowest level of forgiveness and trust (thus, higher level of fear of betrayal and defensiveness). We also contrasted the latter three between themselves.

The strategy preferred by each individual (as Player 1) was identified using the maximum likelihood method. For a given strategy we have calculated which action is expected to be executed. For instance, when following a TFT strategy, we expect that the subject defects (D) in a given round after suffering a defection in the previous one. By considering that for each strategy there is always a well-defined action, we can define a probability function that assigns a high probability $p_H = 0.95$ if the correct (i.e., the expected) action was executed, and a low probability $p_L = 0.05$ otherwise. For instance, in the case of a Coop strategy, we would assign a probability of 0.95 to a cooperation (C) choice and 0.05 to a defection (D) choice, regardless of the outcome of the previous round. In the case of a TFT strategy, we would assign a probability of 0.95 to a cooperation choice following a DC or CC round and to a defection choice following a DD or a CD round; and would assign a probability of 0.05 to the remaining possible outcome combinations. Then, we normalize the resulting likelihood by dividing the un-normalized likelihoods of each strategy by their total sum.

In the end, all of the 30 round-specific likelihood values were applied a logarithmic transformation and then summed, in order to obtain an estimate of how the player's choices across all 30 rounds compare to each of the included strategies.

The un-normalized log-likelihood of a given strategy S given the data D is:

$$l(D|S) = \sum_i \log\left(p_H \delta(a_i|S,D) + p_L(1-\delta(a_i|S,D))\right) \qquad (3)$$

where $\delta(a_i|S,D)$ is 1 if a_i is the expected action for the strategy S and 0 if not.

Given the sequence of all actions, the total likelihood is then the product of the probabilities assigned at each action. Strategies whose actions better correspond to the action chosen by the subject will have higher likelihoods. The strategy exhibiting the highest likelihood according to the game choices of the player was chosen as being that subjects' strategy. The result of this step was an estimate, for each subject, of the most likely strategy adopted during the game. Note that there are two outcomes for each subject because of the two types of partner (computer vs. human), a within-subject effect.

Group-level strategy preference comparisons

For the group analysis, a General Estimating Equation approach was used to estimate a logistic multinomial regression model using a logit link, in R studio. In these models, one of the strategies is used as reference (in this case, TFT), and three model equations are estimated allowing to compare each of the remaining strategies to the reference strategy. Independent between-subject variables were Drug (oxytocin, vasopressin, placebo), and Sex (male, female); and the independent within-subject variable was Partner (human, computer), which entered the model as sets of dummy variables. Again, all main effects and possible interactions were estimated.

In equation (6) a general expression of the equations in this model is presented. Let π_{ijk} be the probability that player *i* chooses strategy *j* with opponent type *k*, i=1,…,n, j=1,…,J and k=1,…,K. Here, in particular, J=4 (i.e. Cooperation, Defection, TFT and TF2T) and K=2 (i.e. human and computer) and the reference profile is a male participant under placebo playing with a human partner.

Given that there are three factors at study here, each of the equations involves a total of 12 parameters covering all the possible combinations (2 genders × 3 drugs × 2 opponent types) as can be seen in Equation 6. Parameters associated to a single variable represent the effects of the variables alone whereas the parameters associated to products of variables represent the effect of the interactions.

$$\ln\left(\frac{\pi_{ijk}}{\pi_{iJk}}\right) = \beta_{0j} + \beta_{1j}Oxytocin_i + \beta_{2j}Vasopressin_i + \beta_{3j}Opponent_{ik} + \beta_{4j}Gender_i + \beta_{5j}Oxytocin_i.Opponent_{ik} + \beta_{6j}Vasopressin_i.Opponent_{ik} + \beta_{7j}Oxytocin_i.Gender_i + \beta_{8j}Vasopressin_i.Gender_i + \beta_{9j}Opponent_{ik}.Gender_i + \beta_{10j}Oxytocin_i.Opponent_{ik}.Gender_i + \beta_{11j}Vasopressin_i.Opponent_{ik}.Gender_i \qquad (6)$$

The variables receiving only one index (i), are those which do not change on the experiment, as it is the case of gender and the dummy variables for the drug given to the participant. Variable '*Opponent* type' receives two indexes since each player faces two opponent types. This way, $Opponent_{ik,}$ k=1,2 represents the types of opponent participant *i* faced.

Each equation allows us to calculate the changes in the log of the chance of preferring strategy *j* to strategy *J* due to changes in the conditions, always comparing the new conditions to the conditions of the "reference" individual – male, receiving a placebo and playing against a human opponent. We calculated 3 equations for each of the 12 profiles. The reference individual corresponds to a zero for all the variables in the model, hence β_{0j} represents the log of the chance that this type of player prefers strategy j over strategy J. By exponentiating β_{0j}, exp(β_{0j}), we get the number of times the probability of strategy *j* being preferred is bigger than the probability that strategy *J* is preferred, for this type of player. Any change in the characteristics of the player cause a change in this relation between the probabilities of preference. For example, if we now consider a female participant (keeping the placebo and the human opponent), the log of the chance is now $\beta_{0j} + \beta_{4j}$ (note that under this profile all other terms of equation 6 will still be zero). The ratio of the probabilities will now be exp($\beta_{0j} + \beta_{4j}$) , which means that the first ratio is altered by a multiplicative factor of exp(β_{4j}) due to the change in the gender of the participant from male to female. Positive values of the betas (exponential of β >1) imply an increase in the ratio of the probabilities, meaning that the presence of the correspondent characteristic favors strategy *j* when compared to strategy *J*.

The set of J-1 equations allows us to compare each strategy with strategy J. The comparison between other pairs of strategies is just a matter of performing some calculations. Also, given that the participants do necessarily chose one of the J strategies,

$$\sum_{j=1}^{J} \pi_{ijk} = 1$$

for every *i* and *j* and hence once the parameters are estimated, all the probabilities π_{ijk} can be obtained after some calculations.

Let η_{ijk} represent the right hand side of equation (6), then

$$\ln\left(\frac{\pi_{ijk}}{\pi_{iJk}}\right) = \eta_{ijk} \leftrightarrow \frac{\pi_{ijk}}{\pi_{iJk}} = e^{\eta_{ijk}} \leftrightarrow \pi_{ijk} = e^{\eta_{ijk}}.\pi_{iJk}$$

Since the probabilities add to one as summed over *j*,

$$\sum_{j=1}^{J} \pi_{ijk} = 1 \leftrightarrow \sum_{j=1}^{J-1} e^{\eta_{ijk}}.\pi_{iJk} + \pi_{iJk} = 1 \leftrightarrow \left(\sum_{j=1}^{J-1} e^{\eta_{ijk}} + 1\right).\pi_{iJk} = 1$$

we get

$$\pi_{iJk} = \frac{1}{\left(\sum_{j=1}^{J-1} e^{\eta_{ijk}} + 1\right)}$$

and consequently

$$\pi_{ijk} = \frac{e^{\eta_{ijk}}}{\left(\sum_{j=1}^{J-1} e^{\eta_{ijk}} + 1\right)}$$

for j=1,…,J-1.

As such, to estimate main effects (of Drug, Partner and Sex), we considered three simple models of the same class as described above, considering each of the independent variables alone. To estimate the 2-way interaction effects (of Drug*Partner, Drug*Sex and Sex*Partner), we considered three models of the same type of equation (6) with all of the possible pairs of factors. We considered a "trend", any effect showing a p-value <0.10, and a "statistically significant effect" any effect showing a p-value <0.05.
