# Supplementary material for: Oxytocin and vasopressin modulation of prisoner’s dilemma strategies
Source: J Psychopharmacol. 2020 Mar 24;34(8):891–900. doi: 10.1177/0269881120913145 (PMC7583454; doi:10.1177/0269881120913145)
Supplement: Supplement_2_JPsychopharm – Supplemental material for Oxytocin and vasopressin modulation of prisoner’s dilemma strategies [file Supplement_2_JPsychopharm.pdf]

Supplement 2 - Results

TFT over TF2T preference

We found a significant preference for TFT over TF2T (p<.05) for all groups, except for 2 groups (where no preference was found between the strategies): 1) female players under placebo playing against a human partner and 2) female players under vasopressin playing against a computer partner (see Figure 1a for preferences and p-values associated with each group). No interactions were found to be statistically significant.

a)

Male	OT	H	2.36 (.017)**	3.00 (.007)**	H	OT	Female
		C	4.00 (.006)**	3.25 (.004)**	C		
	PBO	H	3.88 (<.001)**	1.71 (.109)	H	PBO	
		C	2.22 (.047)**	2.38 (.040)**	C		
	AVP	H	4.57 (<.001)**	3.44 (.001)**	H	AVP	
		C	3.00 (.004)**	1.77 (.100)	C		

b)

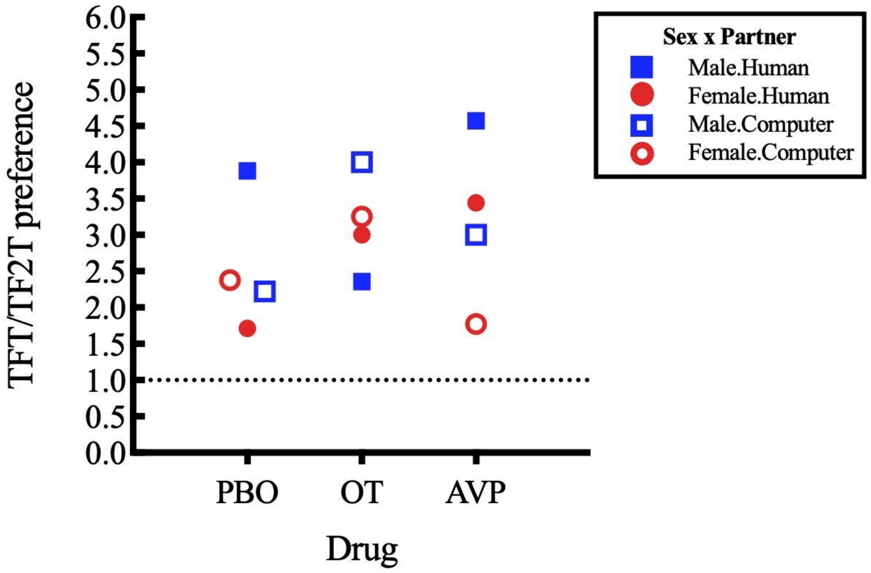

Figure 1. *Preference for Tit-For-Tat (TFT) strategy over Tit-For-Two-Tats (TF2T) strategy as a function of Sex, Drug and Partner. Estimate values >1 present a preference for TFT while estimates <1 point to a preference for TF2T, with p-values marked with ** if statistically*

*significant at p<.05, and with * if a trend at p>.1 in a) table format and b) graphical format. No interactions were found.*

TFT over Def preference

We found a pervasive significant preference (p<.05) for TFT strategy over Def strategy. No interactions emerged as significant (but see Figure 2a for preferences and p-values associated with each group).

Additionally, we found a significant main effect of Partner (p=.029, Table 14), characterized by a 58% drop in the preference for TFT over Def when contrasting a human partner (7.30 times, Table 14) versus a computer partner (7.30 * 0.58=4.23, Table 14).

a)

Male	OT	H	5.20 (<.001)**	8.00 (<.001)**	H	OT	Female
		C	3.33 (.010)**	6.50 (<.001)**	C		
	PBO	H	6.20 (<.001)**	6.00 (<.001)**	H	PBO	
		C	2.86 (.017)**	3.80 (.008)**	C		
	AVP	H	8.00 (<.001)**	15.50 (<.001)**	H	AVP	
		C	5.40 (<.001)**	4.60 (.002)**	C		

b)

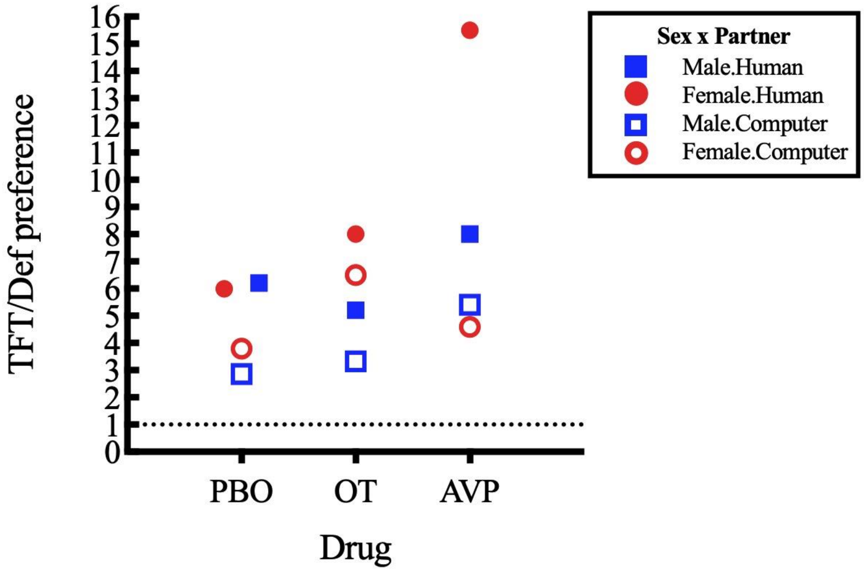

***Figure 2**. Preference for Tit-For-Tat (TFT) strategy over Defector (Def) strategy as a function of Sex, Drug and Partner. Please note the scaling in the y axis. Estimate values >1 present a*

*preference for TFT while estimates <1 point to a preference for Def, with p-values marked with ** if statistically significant at p<.05, and with * if a trend at p<.1, in a) table format and b) graphical format. No significant interactions were found.*

TF2T over Def preference

We found a significant preference for TF2T over Def for female players under placebo playing against a human partner (3.50 times with p=.027). We also found a trend for the same preference for female players under vasopressin, irrespective of partner (4.50 times with p=.054 for a human partner and 2.60 times with p=.069 for a computer partner) (see Figure 3a for preferences and p-values associated with each group). No interactions emerged as significant.

a)

Male	OT	H	2.20 (.144)	2.67 (.147)	H	OT	Female
		C	0.83 (.763)	2.00 (.258)	C		
	PBO	H	1.60 (.410)	3.50 (.027)**	H	PBO	
		C	1.29 (.618)	1.60 (.410)	C		
	AVP	H	1.75 (.372)	4.50 (.054)*	H	AVP	
		C	1.80 (.292)	2.60 (.069)*	C		

b)

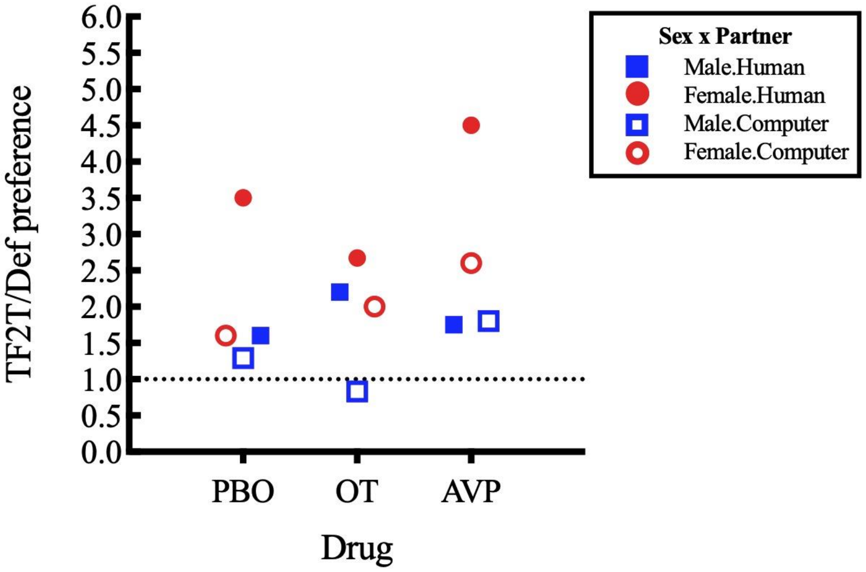

Figure 3. *Preference for Tit-For-Two-Tats (TF2T) strategy over Defector (Def) strategy as a function of Sex, Drug and Partner. Estimate values >1 present a preference for TF2T while estimates <1 point to a preference for Def, with p-values marked with ** if statistically significant*

*at p<.05, and with * if a trend at p<.1, in a) table format and b) graphical format. No significant interactions were found.*

Coop over Def preference

We found a significant preference for Coop over Def for male and female placebo-takers playing against a computer (2.57 times with p=.034 for males and 3.60 times with p=.011 for females). The same preference also emerged for male players under oxytocin playing against a computer partner (3.17 times with p=.014) and for female players under oxytocin playing against a human partner (5.00 times with p=.011). A trend in the same direction emerged for female players under oxytocin playing against a computer (3.00 times with p=.057) and for female players under vasopressin playing against a human partner (4.50 times with p = 0.054) (see Figure 4a for preferences and p-values associated with each group). No interactions emerged as significant.

a)

Male	OT	H	1.60 (.410)	5.00 (.011)**	H	OT	Female
		C	3.17 (.014)**	3.00 (.057)*	C		
	PBO	H	2.00 (.206)	2.00 (.258)	H	PBO	
		C	2.57 (.034)**	3.60 (.011)**	C		
	AVP	H	1.50 (.530)	4.50 (.054)*	H	AVP	
		C	1.60 (.410)	2.00 (.206)	C		

b)

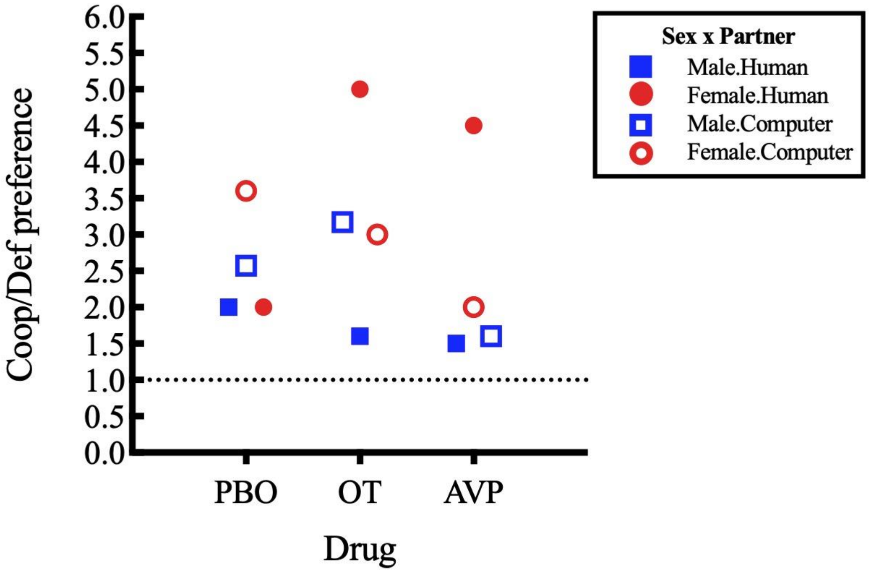

Figure 4. *Preference for Cooperator (Coop) strategy over Defector (Def) strategy as a function of Sex, Drug and Partner. Estimate values >1 present a preference for Coop while estimates <1 point to a preference for Def, with p-values marked with ** if statistically significant at p<.05, and with * if a trend at p<.1, in a) table format and b) graphical format. No significant interactions were found.*

Complete model estimates

Estimates of the effects and interactions on choice of strategy (with corresponding p-values marked with ** if statistically significant at p<0.05, and with * if a trend at p>0.1), of between-subject variables (Sex and Drug) and within-subject variable (Partner type), for each possible reference. Estimate values above 1 present a preference for the strategy in the numerator, while estimates below 1 point to a preference for the denominator strategy.

	Male.Placebo.Human	Oxytocin	Vasopressin	Computer	Female	Oxytocin.Computer	Vasopressin.Computer	Oxytocin.Female	Vasopressin.Female	Computer.Female	Oxytocin.Computer.Female	Vasopressin.Computer.Female
$\frac{\pi_{TFT}}{\pi_{TF2T}}$	3.875 (.001)**	0.601 (.356)	1.180 (.774)	0.573 (.281)	0.442 (.117)	2.951 (.164)	1.144 (.864)	2.869 (.161)	1.703 (.487)	2.416 (.246)	0.265 (.246)	0.324 (.292)
$\frac{\pi_{TFT}}{\pi_{Coop}}$	3.100 (.002)**	1.048 (.931)	1.720 (.345)	0.358 (.019)**	0.968 (.952)	0.904 (.862)	1.766 (.430)	0.509 (.371)	0.667 (.613)	0.982 (.976)	4.259 (.076)*	1.075 (.938)
$\frac{\pi_{TFT}}{\pi_{Def}}$	6.200 (<.001)**	0.839 (.798)	1.290 (.722)	0.461 (.135)	0.968 (.964)	1.391 (.663)	1.465 (.649)	1.590 (.664)	2.002 (.548)	1.374 (.686)	0.922 (.946)	0.320 (.379)
$\frac{\pi_{TF2T}}{\pi_{Coop}}$	0.800 (.638)	1.719 (.415)	1.458 (.606)	0.625 (.384)	2.188 (.228)	0.306 (.172)	1.543 (.661)	0.177 (.057)*	0.392 (.337)	0.406 (.273)	16.073 (.024)**	3.318 (.346)
$\frac{\pi_{TF2T}}{\pi_{Def}}$	1.600 (.410)	1.375 (.685)	1.094 (.916)	0.804 (.701)	2.188 (.330)	0.471 (.434)	1.280 (.809)	0.554 (.617)	1.176 (.900)	0.569 (.523)	3.480 (.393)	0.987 (.993)
$\frac{\pi_{Coop}}{\pi_{Def}}$	2.000 (.206)	0.800 (.778)	0.750 (.734)	1.286 (.636)	1.000 (1.000)	1.539 (.600)	0.830 (.848)	3.125 (.336)	3.000 (.400)	1.400 (.681)	0.217 (.223)	0.298 (.381)

Table 1. Male.Placebo.Human reference

	Male.Placebo.Computer	Oxytocin	Vasopressin	Human	Female	Oxytocin.Human	Vasopressin.Human	Oxytocin.Female	Vasopressin.Female	Human.Female	Oxytocin.Human.Female	Vasopressin.Human.Female
$\frac{\pi_{TFT}}{\pi_{TF2T}}$	2.222 (.047)**	1.800 (.359)	1.350 (.589)	1.744 (.281)	1.069 (.909)	0.339 (0.164)	0.874 (.864)	0.760 (.752)	0.552 (.445)	0.414 (.246)	3.774 (.246)	3.087 (.292)
$\frac{\pi_{TFT}}{\pi_{Coop}}$	1.111 (.746)	0.947 (.906)	3.037 (.032)**	2.790 (.019)**	0.95 (0.912)	1.107 (.862)	0.566 (.430)	2.167 (.243)	0.717 (.645)	1.019 (.976)	0.235 (.076)*	0.930 (.938)
$\frac{\pi_{TFT}}{\pi_{Def}}$	2.857 (.017)**	1.167 (.810)	1.890 (.332)	2.170 (.135)	1.330 (.669)	0.719 (.663)	0.683 (.649)	1.466 (.695)	0.64 (.643)	0.728 (.686)	1.084 (.946)	3.126 (.379)
$\frac{\pi_{TF2T}}{\pi_{Coop}}$	0.500 (.090)*	0.526 (.322)	2.250 (.201)	1.600 (.384)	0.889 (.842)	3.266 (.172)	0.648 (.661)	2.850 (.244)	1.300 (.763)	2.461 (.273)	0.062 (.024)**	0.301 (.346)
$\frac{\pi_{TF2T}}{\pi_{Def}}$	1.286 (.618)	0.648 (.582)	1.400 (.654)	1.244 (.701)	1.244 (.774)	2.121 (.434)	0.781 (.809)	1.929 (.568)	1.161 (.890)	1.758 (.523)	0.287 (.393)	1.013 (.993)
$\frac{\pi_{Coop}}{\pi_{Def}}$	2.571 (.034)**	1.231 (.747)	0.622 (.512)	0.778 (.636)	1.400 (.618)	0.650 (.600)	1.205 (.848)	0.677 (.697)	0.893 (.913)	0.714 (.681)	4.618 (.223)	3.360 (.381)

Table 2. Male.Placebo.Computer reference

	Male.Oxytocin.Human	Placebo	Vasopressin	Computer	Female	Placebo.Computer	Vasopressin.Computer	Placebo.Female	Vasopressin.Female	Computer.Female	Placebo.Computer.Female	Vasopressin.Computer.Female
$\frac{\pi_{TFT}}{\pi_{TF2T}}$	2.364 (.017)**	1.639 (.356)	1.934 (.231)	1.692 (.367)	1.269 (.661)	0.339 (.164)	0.388 (.254)	0.349 (.161)	0.594 (.506)	0.640 (.602)	3.774 (.246)	1.223 (.860)
$\frac{\pi_{TFT}}{\pi_{Coop}}$	3.250 (.004)**	0.954 (.931)	1.641 (.410)	0.324 (.004)**	0.492 (.174)	1.107 (.862)	1.954 (.333)	1.966 (.371)	1.312 (.729)	4.181 (.007)**	0.235 (.076)*	0.252 (.110)
$\frac{\pi_{TFT}}{\pi_{Def}}$	5.200 (.001)**	1.192 (.798)	1.538 (.550)	0.641 (.421)	1.538 (.582)	0.719 (.663)	1.053 (.952)	0.629 (.664)	1.259 (.847)	1.268 (.794)	1.084 (.946)	0.347 (.440)
$\frac{\pi_{TF2T}}{\pi_{Coop}}$	1.375 (.493)	0.582 (.415)	0.848 (.821)	0.191 (.015)**	0.388 (.138)	3.266 (.172)	5.038 (.131)	5.640 (.057)*	2.210 (.413)	6.531 (.041)**	0.062 (.024)**	0.206 (.238)
$\frac{\pi_{TF2T}}{\pi_{Def}}$	2.200 (.144)	0.727 (.685)	0.795 (.782)	0.379 (.209)	1.212 (.824)	2.121 (.434)	2.715 (.384)	1.805 (.617)	2.121 (.570)	1.980 (.556)	0.287 (.393)	0.284 (.442)
$\frac{\pi_{Coop}}{\pi_{Def}}$	1.600 (.410)	1.250 (.778)	0.937 (.940)	1.979 (.277)	3.125 (.181)	0.650 (.600)	0.539 (.549)	0.320 (.336)	0.960 (.975)	0.303 (.210)	4.618 (.223)	1.374 (.828)

Table 3. Male.Oxytocin.Human reference

	Male.Oxytocin.Computer	Placebo	Vasopressin	Human	Female	Placebo.Human	Vasopressin.Human	Placebo.Female	Vasopressin.Female	Human.Female	Placebo.Human.Female	Vasopressin.Human.Female
$\frac{\pi_{TFT}}{\pi_{TF2T}}$	4.000 (.006)**	0.556 (.359)	0.750 (.648)	0.591 (.367)	0.812 (.747)	2.951 0.164)	2.579 (.254)	1.315 (.752)	0.726 (.698)	1.562 (.602)	0.265 (.246)	0.818 (.860)
$\frac{\pi_{TFT}}{\pi_{Coop}}$	1.053 (.873)	1.056 (.906)	3.206 (.024)**	3.087 (.004)**	2.058 (.128)	0.904 (.862)	0.512 (.333)	0.462 (.243)	0.331 (.129)	0.239 (.007)**	4.259 (.076)*	3.962 (.110)
$\frac{\pi_{TFT}}{\pi_{Def}}$	3.333 (.010)**	0.857 (.810)	1.620 (.474)	1.560 (.421)	1.950 (.347)	1.391 (.663)	0.950 (.952)	0.682 (.695)	0.437 (.404)	0.789 (.794)	0.922 (.946)	2.883 (.440)
$\frac{\pi_{TF2T}}{\pi_{Coop}}$	0.263 (.008)**	1.900 (.322)	4.275 (.038)**	5.225 (.015)**	2.533 (.171)	0.306 (.172)	0.198 (.131)	0.351 (.244)	0.456 (.401)	0.153 (.041)**	16.073 (.024)**	4.845 (.238)
$\frac{\pi_{TF2T}}{\pi_{Def}}$	0.833 (.763)	1.543 (.582)	2.160 (.350)	2.640 (.209)	2.400 (.309)	0.471 (.434)	0.368 (.384)	0.519 (.568)	0.602 (.660)	0.505 (.556)	3.480 (.393)	3.525 (.442)
$\frac{\pi_{Coop}}{\pi_{Def}}$	3.167 (.014)**	0.812 (.747)	0.505 (.355)	0.505 (.277)	0.947 (.942)	1.539 (.600)	1.855 (.549)	1.478 (.697)	1.319 (.798)	3.299 (.210)	0.217 (.223)	0.728 (.828)

Table 4. Male.Oxytocin.Computer reference

	Male.Vasopressin.Human	Placebo	Oxytocin	Computer	Female	Placebo.Computer	Oxytocin.Computer	Placebo.Female	Oxytocin.Female	Computer.Female	Placebo.Computer.Female	Oxytocin.Computer.Female
$\frac{\pi_{TFT}}{\pi_{TF2T}}$	4.571 (<.001)**	0.848 (.774)	0.517 (.231)	0.656 (.476)	0.753 (.615)	0.874 (.864)	2.579 (.254)	0.587 (.487)	1.685 (.506)	0.783 (.745)	3.087 (.292)	0.818 (.860)
$\frac{\pi_{TFT}}{\pi_{Coop}}$	5.333 (<.001)**	0.581 (.345)	0.609 (.410)	0.633 (.424)	0.646 (.454)	0.566 (.430)	0.512 (.333)	1.498 (.613)	0.762 (.729)	1.055 (.937)	0.930 (.938)	3.962 (0.110)
$\frac{\pi_{TFT}}{\pi_{Def}}$	8.000 (<.001)**	0.775 (.722)	0.650 (.550)	0.675 (.551)	1.938 (.463)	0.683 (.649)	0.950 (.952)	0.499 (.548)	0.794 (.847)	0.440 (.424)	3.126 (.379)	2.883 (.440)
$\frac{\pi_{TF2T}}{\pi_{Coop}}$	1.167 (.782)	0.686 (.606)	1.179 (.821)	0.964 (.965)	0.857 (.833)	0.648 (.661)	0.198 (.131)	2.552 (.337)	0.453 (.413)	1.348 (.759)	0.301 (.346)	4.845 (.238)
$\frac{\pi_{TF2T}}{\pi_{Def}}$	1.750 (.372)	0.914 (.916)	1.257 (.782)	1.029 (.973)	2.571 (.346)	0.781 (.809)	0.368 (.384)	0.851 (.900)	0.471 (.570)	0.562 (.618)	1.013 (.993)	3.525 (.442)
$\frac{\pi_{Coop}}{\pi_{Def}}$	1.500 (.530)	1.333 (.734)	1.067 (.940)	1.067 (.937)	3.000 (.279)	1.205 (.848)	1.855 (.549)	0.333 (.400)	1.042 (.975)	0.417 (.433)	3.360 (.381)	0.728 (.828)

Table 5. Male.Vasopressin.Human reference

	Male.Vasopressin.Computer	Placebo	Oxytocin	Human	Female	Placebo.Human	Oxytocin.Human	Placebo.Female	Oxytocin.Female	Human.Female	Placebo.Human.Female	Oxytocin.Human.Female
$\frac{\pi_{TFT}}{\pi_{TF2T}}$	3.000 (.004)**	0.741 (.589)	1.333 (.648)	1.524 (.476)	0.590 (.308)	1.144 (.864)	0.388 (.254)	1.812 (.445)	1.378 (.698)	1.278 (.745)	0.324 (.292)	1.223 (.860)
$\frac{\pi_{TFT}}{\pi_{Coop}}$	3.375 (.003)	0.329 (.032)	0.312 (.024)	1.580 (.424)	0.681 (.488)	1.766 (.430)	1.954 (.333)	1.394 (.645)	3.020 (.129)	0.948 (.937)	1.075 (.938)	0.252 (.110)
$\frac{\pi_{TFT}}{\pi_{Def}}$	5.400 (.001)**	0.529 (.332)	0.617 (.474)	1.481 (.551)	0.852 (.817)	1.465 (.649)	1.053 (.952)	1.561 (.643)	2.289 (.404)	2.274 (.424)	0.320 (.379)	0.347 (.440)
$\frac{\pi_{TF2T}}{\pi_{Coop}}$	1.125 (.808)	0.444 (.201)	0.234 (.038)	1.037 (.965)	1.156 (.822)	1.543 (.661)	5.038 (.131)	0.769 (.763)	2.192 (.401)	0.742 (.759)	3.318 (.346)	0.206 (.238)
$\frac{\pi_{TF2T}}{\pi_{Def}}$	1.800 (.292)	0.714 (.654)	0.463 (.350)	0.972 (.973)	1.444 (.632)	1.280 (.809)	2.715 (.384)	0.862 (.890)	1.662 (.669)	1.780 (.618)	0.987 (.993)	0.284 (.442)
$\frac{\pi_{Coop}}{\pi_{Def}}$	1.600 (.410)	1.607 (.512)	1.979 (.355)	0.937 (.937)	1.250 (.778)	0.830 (.848)	0.539 (.549)	1.120 (.913)	0.758 (.798)	2.400 (.433)	0.298 (.381)	1.374 (.828)

Table 6. Male.Vasopressin.Computer reference

	Female.Placebo.Human	Oxytocin	Vasopressin	Computer	Male	Oxytocin.Computer	Vasopressin.Computer	Oxytocin.Male	Vasopressin.Male	Computer.Male	Oxytocin.Computer.Male	Vasopressin.Computer.Male
$\frac{\pi_{TFT}}{\pi_{TF2T}}$	1.714 (.109)	1.750 (.290)	2.009 (.168)	1.385 (.560)	2.260 (.117)	0.782 (.770)	0.371 (.173)	0.349 (.161)	0.587 (.487)	0.414 (.246)	3.774 (.246)	3.087 (.292)
$\frac{\pi_{TFT}}{\pi_{Coop}}$	3.000 (.007)**	0.533 (.231)	1.148 (.804)	0.352 (.019)**	1.033 (.952)	3.849 (.018)**	1.898 (.269)	1.966 (.371)	1.498 (.613)	1.019 (.976)	0.235 (.076)*	0.930 (.938)
$\frac{\pi_{TFT}}{\pi_{Def}}$	6.000 (.001)**	1.333 (.725)	2.583 (.296)	0.633 (.441)	1.033 (.964)	1.283 (.789)	0.469 (.443)	0.629 (.664)	0.499 (.548)	0.728 (.686)	1.084 (.946)	3.126 (.379)
$\frac{\pi_{TF2T}}{\pi_{Coop}}$	1.750 (.207)	0.305 (.056)*	0.571 (.387)	0.254 (.027)**	0.457 (.228)	4.922 (.069)*	5.119 (.042)**	5.640 (.057)*	2.552 (.337)	2.461 (.273)	0.062 (.024)**	0.301 (.346)
$\frac{\pi_{TF2T}}{\pi_{Def}}$	3.500 (.027)**	0.762 (.758)	1.286 (.795)	0.457 (.247)	0.457 (.330)	1.641 (.652)	1.264 (.821)	1.805 (.617)	0.851 (.900)	1.758 (.523)	0.287 (.393)	1.013 (.993)
$\frac{\pi_{Coop}}{\pi_{Def}}$	2.000 (.258)	2.500 (.298)	2.250 (.414)	1.800 (.346)	1.000 (1.000)	0.333 (.247)	0.247 (.155)	0.320 (.336)	0.333 (.400)	0.714 (.681)	4.618 (.223)	3.360 (.381)

Table 7. Female.Placebo.Human reference

	Female.Placebo.Computer	Oxytocin	Vasopressin	Human	Male	Oxytocin.Human	Vasopressin.Human	Oxytocin.Male	Vasopressin.Male	Human.Male	Oxytocin.Human.Male	Vasopressin.Human.Male
$\frac{\pi_{TFT}}{\pi_{TF2T}}$	2.375 (.040)**	1.368 (.591)	0.745 (.590)	0.722 (.560)	0.936 (.909)	1.279 (.770)	2.697 (.173)	1.315 (.752)	1.812 (.445)	2.416 (.246)	0.265 (.246)	0.324 (.292)
$\frac{\pi_{TFT}}{\pi_{Coop}}$	1.056 (.869)	2.053 (.134)	2.179 (.121)	2.842 (.019)**	1.053 (.912)	0.260 (.018)**	0.527 (.269)	0.462 (.243)	1.394 (.645)	0.982 (.976)	4.259 (.076)*	1.075 (.938)
$\frac{\pi_{TFT}}{\pi_{Def}}$	3.800 (.008)**	1.711 (.466)	1.211 (.786)	1.579 (.441)	0.752 (.669)	0.779 (.789)	2.134 (.443)	0.682 (.695)	1.561 (.643)	1.374 (.686)	0.922 (.946)	0.320 (.379)
$\frac{\pi_{TF2T}}{\pi_{Coop}}$	0.444 (.056)*	1.500 (.516)	2.925 (.073)*	3.938 (.027)**	1.125 (.842)	0.203 (.069)*	0.195 (.042)**	0.351 (.244)	0.769 (.763)	0.406 (.273)	16.073 (.024)**	3.318 (.346)
$\frac{\pi_{TF2T}}{\pi_{Def}}$	1.600 (.410)	1.250 (.790)	1.625 (.531)	2.188 (.247)	0.804 (.774)	0.610 (.652)	0.791 (.821)	0.519 (.568)	0.862 (.890)	0.569 (.523)	3.48 (.393)	0.987 (.993)
$\frac{\pi_{Coop}}{\pi_{Def}}$	3.600 (.011)**	0.833 (.812)	0.556 (.430)	0.556 (.346)	0.714 (.618)	3.00 (.247)	4.050 (.155)	1.478 (.697)	1.120 (.913)	1.400 (.681)	0.217 (.223)	0.298 (.381)

Table 8. Female.Placebo.Computer reference

	Female.Oxytocin.Human	Placebo	Vasopressin	Computer	Male	Placebo.Computer	Vasopressin.Computer	Placebo.Male	Vasopressin.Male	Computer.Male	Placebo.Computer.Male	Vasopressin.Computer.Male
$\frac{\pi_{TFT}}{\pi_{TF2T}}$	3.000 (.007)**	0.571 (.290)	1.148 (.804)	1.083 (.898)	0.788 (.661)	1.279 (.770)	0.474 (.339)	2.869 (.161)	1.685 (.506)	1.562 (.602)	0.265 (.246)	0.818 (.860)
$\frac{\pi_{TFT}}{\pi_{Coop}}$	1.600 (.153)	1.875 (.231)	2.153 (.126)	1.354 (.396)	2.031 (.174)	0.260 (.018)**	0.493 (.170)	0.509 (.371)	0.762 (.729)	0.239 (.007)**	4.259 (.076)*	3.962 (.110)
$\frac{\pi_{TFT}}{\pi_{Def}}$	8.000 (.001)**	0.750 (.725)	1.938 (.487)	0.812 (.773)	0.650 (.582)	0.779 (.789)	0.365 (.345)	1.590 (.664)	0.794 (.847)	0.789 (.794)	0.922 (.946)	2.883 (.440)
$\frac{\pi_{TF2T}}{\pi_{Coop}}$	0.533 (.151)	3.281 (.056)*	1.875 (.329)	1.250 (.720)	2.578 (.138)	0.203 (.069)*	1.040 (.961)	0.177 (.057)*	0.453 (.413)	0.153 (.041)**	16.073 (.024)**	4.845 (.238)
$\frac{\pi_{TF2T}}{\pi_{Def}}$	2.667 (.147)	1.312 (.758)	1.688 (.613)	0.750 (.740)	0.825 (.824)	0.610 (.652)	0.770 (.824)	0.554 (.617)	0.471 (.570)	0.505 (.556)	3.480 (.393)	3.525 (.442)
$\frac{\pi_{Coop}}{\pi_{Def}}$	5.000 (.011)**	0.400 (.298)	0.900 (.917)	0.600 (.475)	0.320 (.181)	3.000 (.247)	0.741 (.774)	3.125 (.336)	1.042 (.975)	3.299 (.210)	0.217 (.223)	0.728 (.828)

Table 9. Female.Oxytocin.Human reference

	Female.Oxytocin.Computer	Placebo	Vasopressin	Human	Male	Placebo.Human	Vasopressin.Human	Placebo.Male	Vasopressin.Male	Human.Male	Placebo.Human.Male	Vasopressin.Human.Male
$\frac{\pi_{TFT}}{\pi_{TF2T}}$	3.250 (.004)**	0.731 (.591)	0.544 (.254)	0.923 (.898)	1.231 (.747)	0.782 (.770)	2.109 (.339)	0.760 (.752)	1.378 (.698)	0.640 (.602)	3.774 (.246)	1.223 (.860)
$\frac{\pi_{TFT}}{\pi_{Coop}}$	2.167 (.027)**	0.487 (.134)	1.062 (.908)	0.738 (.396)	0.486 (.128)	3.849 (.018)**	2.028 (.170)	2.167 (.243)	3.020 (.129)	4.181 (.007)**	0.235 (.076)*	0.252 (.110)
$\frac{\pi_{TFT}}{\pi_{Def}}$	6.500 (<.001)**	0.585 (.466)	0.708 (.635)	1.231 (.773)	0.513 (.347)	1.283 (.789)	2.738 (.345)	1.466 (.695)	2.289 (.404)	1.268 (.794)	1.084 (.946)	0.347 (.440)
$\frac{\pi_{TF2T}}{\pi_{Coop}}$	0.667 (.374)	0.667 (.516)	1.950 (.282)	0.800 (.720)	0.395 (.171)	4.922 (.069)*	0.962 (.961)	2.850 (.244)	2.192 (.401)	6.531 (.041)**	0.062 (.024)**	0.206 (.238)
$\frac{\pi_{TF2T}}{\pi_{Def}}$	2.000 (.258)	0.800 (.790)	1.300 (.745)	1.333 (.740)	0.417 (.309)	1.641 (.652)	1.298 (.824)	1.929 (.568)	1.662 (.660)	1.980 (.556)	0.287 (.393)	0.284 (.442)
$\frac{\pi_{Coop}}{\pi_{Def}}$	3.000 (.057)*	1.200 (.812)	0.667 (.610)	1.667 (.475)	1.056 (.942)	0.333 (.247)	1.350 (.774)	0.677 (.697)	0.758 (.798)	0.303 (.210)	4.618 (.223)	1.374 (.828)

Table 10. Female.Oxytocin.Computer reference

	Female.Vasopressin.Human	Placebo	Oxytocin	Computer	Male	Placebo.Computer	Oxytocin.Computer	Placebo.Male	Oxytocin.Male	Computer.Male	Placebo.Computer.Male	Oxytocin.Computer.Male
$\frac{\pi_{TFT}}{\pi_{TF2T}}$	3.444 (.001)**	0.498 (.168)	0.871 (.804)	0.514 (.152)	1.327 (.615)	2.697 (.173)	2.109 (.339)	1.703 (.487)	0.594 (.506)	1.278 (.745)	0.324 (.292)	1.223 (.860)
$\frac{\pi_{TFT}}{\pi_{Coop}}$	3.444 (.001)**	0.871 (.804)	0.465 (.126)	0.668 (.277)	1.548 (.454)	0.527 (.269)	2.028 (.170)	0.667 (.613)	1.312 (.729)	0.948 (.937)	1.075 (.938)	0.252 (.110)
$\frac{\pi_{TFT}}{\pi_{Def}}$	15.500 (<.001)**	0.387 (.296)	0.516 (.487)	0.297 (.124)	0.516 (.463)	2.134 (.443)	2.738 (.345)	2.002 (.548)	1.259 (.847)	2.274 (.424)	0.320 (.379)	0.347 (.440)
$\frac{\pi_{TF2T}}{\pi_{Coop}}$	1.000 (1.000)	1.750 (.387)	0.533 (.329)	1.300 (.606)	1.167 (.833)	0.195 (.042)**	0.962 (.961)	0.392 (.337)	2.210 (.413)	0.742 (.759)	3.318 (.346)	0.206 (.238)
$\frac{\pi_{TF2T}}{\pi_{Def}}$	4.500 (.054)*	0.778 (.795)	0.593 (.613)	0.578 (.486)	0.389 (.346)	0.791 (.821)	1.298 (.824)	1.176 (.900)	2.121 (.570)	1.780 (.618)	0.987 (.993)	0.284 (.442)
$\frac{\pi_{Coop}}{\pi_{Def}}$	4.500 (.054)*	0.444 (.414)	1.111 (.917)	0.444 (.286)	0.333 (.279)	4.050 (.155)	1.350 (.774)	3.000 (.400)	0.960 (.975)	2.400 (.433)	0.298 (.381)	1.374 (.828)

Table 11. Female.Vasopressin.Human reference

	Female.Vasopressin.Computer	Placebo	Oxytocin	Human	Male	Placebo.Human	Oxytocin.Human	Placebo.Male	Oxytocin.Male	Human.Male	Placebo.Human.Male	Oxytocin.Human.Male
$\frac{\pi_{TFT}}{\pi_{TF2T}}$	1.769 (.100)	1.342 (.590)	1.837 (.254)	1.947 (.152)	1.696 (.308)	0.371 (.173)	0.474 (.339)	0.552 (.445)	0.726 (.698)	0.783 (.745)	3.087 (.292)	0.818 (.860)
$\frac{\pi_{TFT}}{\pi_{Coop}}$	2.300 (.028)**	0.459 (.121)	0.942 (.908)	1.498 (.277)	1.467 (.488)	1.898 (.269)	0.493 (.170)	0.717 (.645)	0.331 (.129)	1.055 (.937)	0.930 (.938)	3.962 (.110)
$\frac{\pi_{TFT}}{\pi_{Def}}$	4.600 (.002)**	0.826 (.786)	1.413 (.635)	3.370 (.124)	1.174 (.817)	0.469 (.443)	0.365 (.345)	0.640 (.643)	0.437 (.404)	0.440 (.424)	3.126 (.379)	2.883 (.440)
$\frac{\pi_{TF2T}}{\pi_{Coop}}$	1.300 (.533)	0.342 (.073)*	0.513 (.282)	0.769 (.606)	0.865 (.822)	5.119 (.042)**	1.040 (.961)	1.300 (.763)	0.456 (.401)	1.348 (.759)	0.301 (.346)	4.845 (.238)
$\frac{\pi_{TF2T}}{\pi_{Def}}$	2.600 (.069)*	0.615 (.531)	0.769 (.745)	1.731 (.486)	0.692 (.632)	1.264 (.821)	0.770 (.824)	1.161 (.809)	0.602 (.660)	0.562 (.618)	1.013 (.993)	3.525 (.442)
$\frac{\pi_{Coop}}{\pi_{Def}}$	2.000 (.206)	1.800 (.430)	1.500 (.610)	2.250 (.286)	0.800 (.778)	0.247 (.155)	0.741 (.774)	0.893 (.913)	1.319 (.798)	0.417 (.433)	3.360 (.381)	0.728 (.828)

Table 12. Female.Vasopressin.Computer reference

Main Effects

Estimates of main effects on choice of strategy (with corresponding p-values marked with ** if statistically significant at $p<0.05$, and with * if a trend at $p<0.1$), of between-subject variables (Sex and Drug) and within-subject variable Partner type. Estimate values above 1 present a preference for the strategy in the numerator, while estimates below 1 point to a preference for the denominator strategy.

	Male	Female
$\frac{\pi_{TFT}}{\pi_{TF2T}}$	3.184 (<.001)**	0.770 (.248)
$\frac{\pi_{TFT}}{\pi_{Coop}}$	2.261 (<.001)**	0.903 (.664)
$\frac{\pi_{TFT}}{\pi_{Def}}$	4.875 (<.001)**	1.311 (.417)
$\frac{\pi_{TF2T}}{\pi_{Coop}}$	0.710 (.067)*	1.173 (.540)
$\frac{\pi_{TF2T}}{\pi_{Def}}$	1.531 (.086)*	1.704 (.150)
$\frac{\pi_{Coop}}{\pi_{Def}}$	3.156 (.002)**	1.452 (.313)

Table 13. Main effect of Sex with Male as a reference

	Human	Computer
$\frac{\pi_{\mathrm{TFT}}}{\pi_{\mathrm{TF2T}}}$	2.947 (<.001)**	0.881 (.566)
$\frac{\pi_{\mathrm{TFT}}}{\pi_{Coop}}$	3.000 (<.001)**	0.529 (<.001)**
$\frac{\pi_{\mathrm{TFT}}}{\pi_{\mathrm{Def}}}$	7.304 (<.001)**	0.578 (.029)**
$\frac{\pi_{\mathrm{TF2T}}}{\pi_{Coop}}$	1.018 (.925)	0.601 (.041)**
$\frac{\pi_{\mathrm{TF2T}}}{\pi_{\mathrm{Def}}}$	2.478 (<.001)**	0.656 (.151)
$\frac{\pi_{\mathrm{Coop}}}{\pi_{\mathrm{Def}}}$	2.425 (<.001)**	1.091 (.742)

Table 14. Main effect of Partner with Human as a reference

	Placebo	Oxytocin	Vasopressin
$\frac{\pi_{\mathrm{TFT}}}{\pi_{\mathrm{TF2T}}}$	2.410 (<.001)**	1.245 (.431)	1.234 (.442)
$\frac{\pi_{\mathrm{TFT}}}{\pi_{Coop}}$	1.741 (.003)**	1.021 (.939)	1.967 (.022)
$\frac{\pi_{\mathrm{TFT}}}{\pi_{\mathrm{Def}}}$	4.476 (<.001)**	1.191 (.664)	1.578 (.256)
$\frac{\pi_{\mathrm{TF2T}}}{\pi_{Coop}}$	0.722 (.127)	0.821 (.519)	1.594 (.155)
$\frac{\pi_{\mathrm{TF2T}}}{\pi_{\mathrm{Def}}}$	1.857 (.049)**	0.957 (.921)	1.279 (.584)
$\frac{\pi_{\mathrm{Coop}}}{\pi_{\mathrm{Def}}}$	2.571 (.002)	1.167 (.723)	0.802 (.631)

Table 15. Main effect of Drug with Placebo as a reference
